# Sex Differences in Correlates of Intermediate Phenotypes and Prevalent Cardiovascular Disease in the General Population

**DOI:** 10.3389/fcvm.2015.00015

**Published:** 2015-04-15

**Authors:** Renate B. Schnabel, Philipp S. Wild, Jürgen H. Prochaska, Francisco M. Ojeda, Tanja Zeller, Nargiz Rzayeva, Ariana Ebrahim, Karl J. Lackner, Manfred E. Beutel, Norbert Pfeiffer, Christoph R. Sinning, Sabine Oertelt-Prigione, Vera Regitz-Zagrosek, Harald Binder, Thomas Münzel, Stefan Blankenberg, for the Gutenberg Health Study Investigators

**Affiliations:** ^1^Department of General and Interventional Cardiology, University Heart Center Hamburg-Eppendorf, Hamburg, Germany; ^2^Center for Thrombosis and Hemostasis, University Medical Center Mainz, Mainz, Germany; ^3^Department of Medicine 2, University Medical Center Mainz, Mainz, Germany; ^4^German Centre for Cardiovascular Research (DZHK), Partner Site RheinMain, Germany; ^5^Institute of Clinical Chemistry and Laboratory Medicine, University Medical Center Mainz, Mainz, Germany; ^6^Department of Psychosomatic Medicine and Psychotherapy, University Medical Center Mainz, Mainz, Germany; ^7^Department of Ophthalmology, University Medical Center Mainz, Mainz, Germany; ^8^Institute of Gender in Medicine (GiM), Center for Cardiovascular Research (CCR), Charité-Universitätsmedizin Berlin, Berlin, Germany; ^9^German Centre for Cardiovascular Research (DZHK), Partner Site Berlin, Germany; ^10^Institute of Medical Biostatistics, Epidemiology and Informatics (IMBEI), University Medical Center Mainz, Mainz, Germany

**Keywords:** sex differences, non-invasive vascular function, cardiac function, population-based study, cardiovascular epidemiology

## Abstract

**Background:**

There are marked sex differences in cardiovascular disease (CVD) manifestation. It is largely unknown how the distribution of CVD risk factors or intermediate phenotypes explain sex-specific differences.

**Methods and Results:**

In 5000 individuals of the population-based Gutenberg Health Study, mean age 55 ± 11 years, 51% males, we examined sex-specific associations of classical CVD risk factors with intima-media thickness, ankle-brachial index, flow-mediated dilation, peripheral arterial tonometry, echocardiographic, and electrocardiographic variables. Intermediate cardiovascular phenotypes were related to prevalent CVD [coronary artery disease, heart failure, stroke, myocardial infarction, lower extremity artery disease (LEAD) *N* = 561].

We observed differential distributions of CVD risk factors with a higher risk factor burden in men. Manifest coronary artery disease, stroke, myocardial infarction, and LEAD were more frequent in men; the proportion of heart failure was higher in women. Intermediate phenotypes showed clear sex differences with more beneficial values in women. Fairly linear changes toward less beneficial values with age were observed in both sexes. In multivariable-adjusted regression analyses, age, systolic blood pressure, and body mass index were consistently associated with intermediate phenotypes in both sexes with different ranking according to random forests, maximum model R^2^ 0.43. Risk factor-adjusted associations with prevalent CVD showed some differences by sex. No interactions by menopausal status were observed.

**Conclusion:**

In a population-based cohort, we observed sex differences in risk factors and a broad range of intermediate phenotypes of non-invasive cardiovascular structure and function. Their relation to prevalent CVD differed markedly. Our results indicate the need of future investigations to understand sex differences in CVD manifestation.

## Introduction

Cardiovascular diseases (CVDs) are the leading cause of death worldwide in both sexes (1). As data in the United States show, the absolute number of women living with or dying of CVD is higher compared to men (2), although women develop CVD such as myocardial infarction on average 10 years later than their male counterparts (3). Sex differences in common CVD manifestations such as coronary heart disease, stroke, heart failure, sudden cardiac death, and lower extremity artery disease have long been known (4–6). Whereas hospitalizations for coronary heart disease are more frequent in men, stroke and heart failure are more prevalent in women hospitalized for CVD (2). Although the underlying pathophysiological process of these epidemic diseases is fundamentally similar, differences in inflammatory activity in the coronary arteries and plaque development are evident (7). Women show a more diffuse atherosclerosis pattern and eccentric remodeling. The transition toward vulnerable plaques is slower in females (8). The clinical manifestations also differ. Prior literature suggests that sex differences cannot be fully explained by the distribution of classical CVD risk factors alone (4, 9). Intermediate phenotypes in the pathophysiological pathway of cardiovascular disease may help to better understand susceptibility, early stages, and progression of disease. In this context, sex differences have been reported for vascular function assessed by intima-media thickness (IMT) and ankle-brachial index (ABI), (10) or flow-mediated dilation (FMD) and pulse amplitude (11, 12). Left ventricular geometry and function is correlated with sex (13), and sex-specific electrocardiographic (ECG) parameters have been described (14). All of these cardiovascular function measures have been related to cardiovascular risk factor burden. Differences in cardiovascular risk factor associations with disease by sex have been established. In particular, diabetes and smoking are known for their stronger impact on cardiovascular disease risk in women (15, 16). Whether sex differences in distribution of classical cardiovascular risk factors may help to explain observed differences across intermediate phenotypes and prevalent CVD is unknown. Data truly representative of the general population are rare (6). The aim of our study therefore was to explore sex differences and cardiovascular risk factor correlates of continuous intermediate phenotypes of (1) non-invasive structure and function of different vascular beds by ultrasound and pulse amplitude measurement, and (2) the myocardium by ECG and echocardiography in relation to prevalence of CVD in a contemporary cohort of middle-aged population-based individuals.

## Materials and Methods

### Study sample

Current analyses comprise the first 5000 individuals of the population-based Gutenberg Health Study. It constitutes a cohort of European descent enrolled from 2007 on at the Department of Medicine 2, University Medical Center Mainz, randomly selected through the local registry office from the city of Mainz and the region Mainz-Bingen. Participants were aged between 35 and 74 years. Enrollment was stratified by age decade and sex, so that 50% of invited individuals were women (12). The response rate of the study was 67%. During their clinic visit, a comprehensive information on cardiovascular risk factors and menopausal status was collected by standardized computer-assisted interview, anthropometric measures, and non-invasive cardiovascular function testing. Smoking status was summarized in two categories: non-smokers (never smokers and former smokers) and smokers. Diabetes mellitus was defined as a physician diagnosis of diabetes and/or a fasting blood glucose concentration measured on site of ≥126 mg/dL (minimum 8-h fast) or a blood glucose level of ≥200 mg/dL at any time. Dyslipidemia was diagnosed when the participant reported a physician’s diagnosis of dyslipidemia and/or an LDL/HDL ratio of >3.5 were determined. Hypertension comprised anti-hypertensive drug treatment and/or a mean systolic blood pressure of ≥140 mmHg and/or a mean diastolic blood pressure of ≥90 mmHg. Socioeconomic status was assessed by Lampert’s and Kroll’s Score (17).

A family history of myocardial infarction was defined as the age at myocardial infarction of a male first-degree relative ≤60 years and/or of a female first-degree relative ≤65 years. Further, self-reported coronary artery disease, heart failure, a history of myocardial infarction, stroke, and/or lower extremity artery disease were assessed. The diagnosis of symptomatic heart failure comprised a NYHA classification ≥II, treatment of heart failure within the last 12 months, and echocardiographically determined left ventricular ejection fraction <55%, and/or diastolic dysfunction (18).

### Intermediate phenotypes

We chose distinct non-invasively determined intermediate phenotypes that indicate changes in cardiovascular structure and function and have been related to cardiac aging and CVD.

Intima-media thickness and ABI reflect central and peripheral vascular structure; FMD and peripheral pulse amplitude correlate with conductance and peripheral artery function, FMD in part with endothelial reactivity. Left ventricular mass and ejection fraction mirror cardiac structure and function complemented by ECG phenotypes of atrial and ventricular conductance.

Intima-media thickness was determined by an 11- to 3-MHz linear array transducer on an iE33 ultrasound system (Philips Medical Systems). Online R-wave triggered measurements were performed using commercially available automated computerized system (QLAB; Philips Medical Systems). Far wall IMT was measured in 1 cm distance of the carotid bulb over a length of 1 cm. IMT is presented as the mean IMT, which is the mean value of right and left IMT measurements.

For ABI, systolic blood pressure was measured at the left arm with an Omron HEM 705-CP II device. Peripheral pressure was taken using a hand-held 8-MHz Doppler probe (handydop, Elcat) and an aneroid sphygmomanometer at the posterior tibial artery at both ankles. The mean value was determined.

Brachial artery diameters were registered at the right brachial artery on a HD11XE CV ultrasound system by an L12–5 (38 mm) linear array broadband probe (Philips Medical Systems, Best, The Netherlands). FMD was determined offline using a commercially available software package (Medical Imaging Applications LLC) and expressed as a relative change from baseline (([60-second diameter]-[baseline diameter])/baseline diameter)*100 = FMD%. Peripheral arterial tonometry (PAT) was measured by pneumatic pulse amplitude recorded at the right index finger by the Endo-PAT2000 device (Itamar Medical, Caesarea, Israel) in parallel with FMD measurements. A log-transformed measure of peripheral pulse amplitude (arbitrary units) is given.

Echocardiography was performed on an iE33 ultrasound system using an S5-1 sector array transducer (Philips Medical Systems, Best, The Netherlands). Left ventricular mass, planimetric ejection fraction, and E/E’ (with E’ assessed from the lateral mitral annulus) as a continuous variable of diastolic function were analyzed according to current guidelines (19).

A 12-lead surface ECG was registered (GE Cardiosoft^®^, GE Healthcare, Germany) and automated measurements were transferred to the data set. All ECGs were reviewed by two cardiologists. Details on the measurement of the intermediate phenotypes in the Gutenberg Health Study have been published earlier (10, 12, 13, 20).

The study has been approved by the Local Ethics Committee; prior to enrollment, study participants provided written, informed consent. All authors have read and approved the manuscript as written.

### Statistical methods

We analyzed data in all available individuals (*N* = 5000) stratified by sex. To achieve near-normal distribution of continuous phenotype variables, we log-transformed IMT, baseline pulse amplitude, left ventricular wall mass, PR interval, and E/E’. FMD was square-root transformed after subtracting its minimum to guarantee that all values were non-negative; this transformation was chosen after examining the results of the Box-Cox transformation applied separately to each gender. The distribution (median, 25th, 75th) of intermediate phenotypes according to age groups was computed using quantile regression and modeling age via quadratic B-splines.

We ran separate age- and multivariable-adjusted linear regression analyses with intermediate phenotypes [IMT, ABI, brachial artery diameter, FMD, baseline pulse amplitude, PAT ratio, left ventricular ejection fraction and wall mass, electrocardiographic PR, and heart rate corrected QT duration (QTc)] as outcome. The multivariable models were adjusted for classical CVD risk factors, such as age, smoking status, systolic blood pressure, diabetes mellitus, dyslipidemia, family history of myocardial infarction, and body mass index (BMI). Since we hypothesized sex differences of non-invasive vascular function measures in manifest disease, multivariable-adjusted logistic regressions were further performed for intermediate phenotypes in relation to manifest CVD (coronary artery disease, heart failure, stroke, myocardial infarction, LEAD). Odds ratios (OR) with 95% confidence interval (CI) were calculated. To test for sex interactions, the aforementioned linear and logistic regressions were also computed without sex stratification. These models were adjusted for sex and included all possible sex interactions.

For all regressions involving FMD, additional models were calculated, where, following the recommendations to account for possible differences in baseline diameters, they were further adjusted for this variable (21).

To understand the relative importance and possible interactions of classical CVD risk factors in relation to intermediate phenotypes by sex, a random forest was calculated for each cardiovascular phenotype (22) with the risk factors as predictors. The accuracy measure used was the mean squared error (MSE). In our analyses, we produced 2500 trees per forest. Details on the method are provided in the Supplementary Material.

We assumed *P* < 0.05 as the threshold for statistical significance. Analyses were exploratory in nature.

### Secondary analyses

To account for possible differences in hypertension treatment and socioeconomic status by sex, we performed an additional multivariable-adjusted model including hypertension medication and socioeconomic status, respectively.

To assess potential interactions by menopause in associations in women, we included interaction terms for menopausal status. In addition, a sub-analyses in women younger than 51 [age-threshold is the 95th age-percentile in females, who declared to have regular menstruations (extreme outliers in this group were excluded from analyses)] were performed.

For statistical calculations, we used R software, Version 3.0.2 (R Core Team, 2013. R: A language and environment for statistical computing. R Foundation for Statistical Computing, Vienna, Austria).

## Results

Sex-specific characteristics of the sample are shown in Table 1. Due to the study design, sex was equally distributed and the mean age was 56 ± 11 years in men and 55 ± 11 years in women. Except for a family history of myocardial infarction, the risk factor profile of men revealed more adverse CVD risk factors compared to women, with a higher proportion of current smokers, diabetes, hypertension, and dyslipidemia. BMI and systolic blood pressure were higher in males on average. Prevalent CVD compromising coronary artery disease, myocardial infarction, stroke, and LEAD were more frequent in men, whereas heart failure appeared to be more often diagnosed in women.

**Table 1:** **Characteristics of the study sample by sex**.

Variable	Men	Women
	*N* = 2540	*N* = 2460
**Cardiovascular risk factors**
Age (years)	56 (46, 66)	55 (45, 64)
Current smoking, *n* (%)	527 (20.8)	432 (17.6)
Diabetes, *n* (%)	247 (9.7)	127 (5.2)
Hypertension, *n* (%)	1426 (56.1)	1138 (46.3)
Family history of MI, *n* (%)	433 (17)	453 (18.4)
Dyslipidemia, *n* (%)	923 (36.4)	539 (21.9)
BMI (kg/m^2^)	27.2 (24.9, 30)	25.6 (22.9, 29.5)
Systolic blood pressure (mmHg)	134 (124, 145)	128 (116, 141)
Antihypertensive drugs, *n* (%)	838 (33.1)	721 (29.4)
**Cardiovascular diseases**
Coronary artery disease, *n* (%)	173 (6.9)	53 (2.2)
Heart failure, *n* (%)	125 (5.1)	134 (5.6)
Stroke, *n* (%)	59 (2.3)	36 (1.5)
Myocardial infarction, *n* (%)	119 (4.7)	37 (1.5)
LEAD, *n* (%)	109 (4.3)	94 (3.8)
**Biomarker**
HDL cholesterol (mg/dL)	48 (40.6, 56.6)	62 (52.6, 73.0)
C-reactive protein (mg/L)	1.6 (0.5, 3)	1.7 (1, 3.7)
**Socioeconomic status**
Socioeconomic status	13 (10, 17)	11 (9, 15)
**Intermediate phenotypes**
Intima-media thickness (mm)	0.65 (0.56, 0.75)	0.62 (0.55, 0.7)
Ankle-brachial index	1 (0.92, 1.06)	0.98 (0.91, 1.04)
Baseline brachial artery diameter (mm)	4.9 (4.5, 5.3)	3.7 (3.3, 4.1)
Flow-mediated dilation (%)	5.99 (3.77, 8.62)	8.97 (5.58, 13.04)
Baseline pulse amplitude	6.55 (5.97, 6.99)	5.72 (5.05, 6.37)
PAT ratio	0.4 (0.2, 0.8)	0.8 (0.5, 1)
Left ventricular ejection fraction (%)	63.5 (59.6, 67.5)	64.7 (61.1, 68.2)
Left ventricular wall mass (g)	176 (152, 206)	128 (108, 152)
E/E’	6.8 (5.55, 8.57)	7.16 (5.92, 8.8)
PR interval (ms)	162 (148, 178)	154 (138, 168)
QTc Duration (ms)	413 (398, 430)	424 (411, 439)

*Data presented are median (25th, 75th percentile) for continuous variables or number (%) for discrete variables*.

*BMI, body mass index; LEAD, lower extremity artery disease; PAT, peripheral arterial tonometry*.

Intermediate phenotypes tended to show more beneficial characteristics in women in comparison with their male counterparts. In women, mean IMT, baseline brachial artery diameter, ABI, left ventricular wall mass, ECG duration of PR interval were lower, whereas FMD, PAT ratio, QTc, and left ventricular ejection fraction as well as E/E’ tended to be higher. In Figure S1 in Supplementary Material, the distribution of intermediate phenotypes is displayed as boxplots. The distribution of intermediate phenotypes by age showed a general trend toward less beneficial values with advancing age across all phenotypes and in both sexes, except for PAT ratio and ABI, which showed an inverse U shaped association (Figure S2 in Supplementary Material). Development with age appeared to be close to linear for most intermediate phenotypes.

### Classical CVD risk factors in relation to intermediate phenotypes

**Figure 1:**
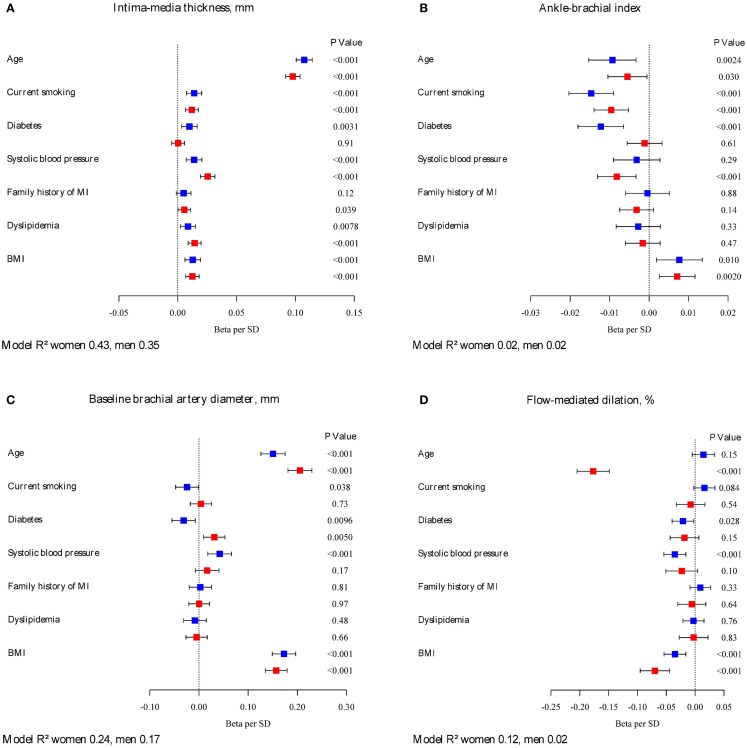

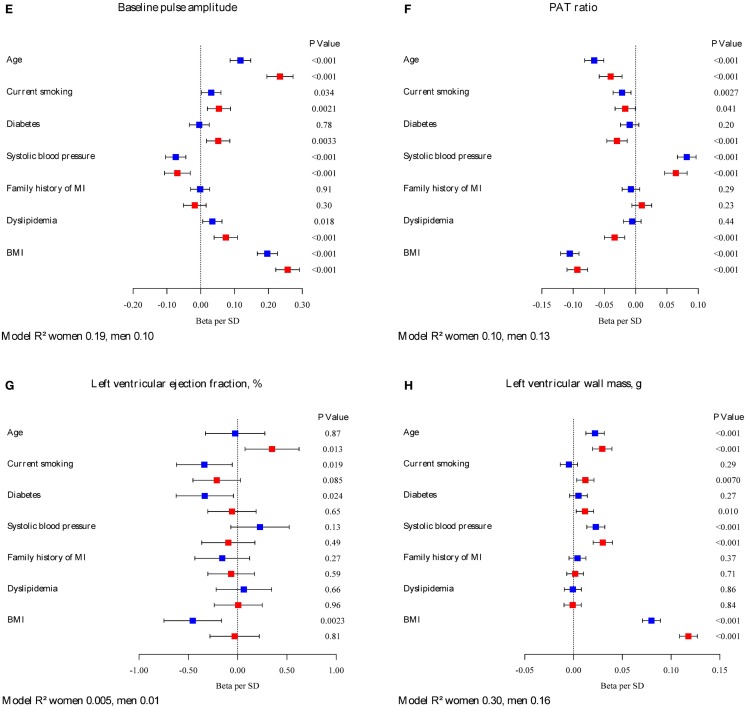

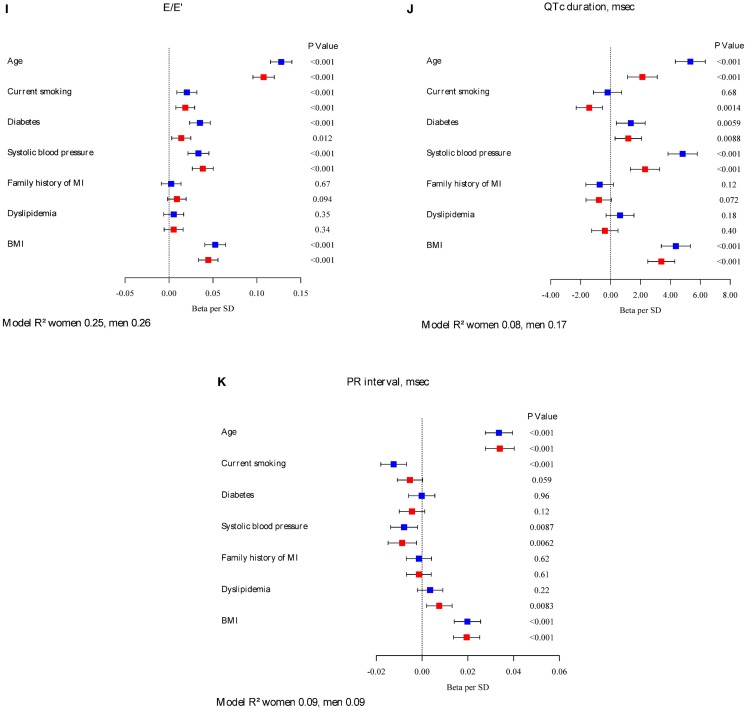
**Betas per SD increase and 95% confidence intervals for classical CVD risk factors in relation to intermediate phenotypes by sex**. Continuous variables of intermediate phenotypes are **(A)** intima-media thickness, **(B)** ankle-brachial index, **(C)** baseline brachial artery diameter, **(D)** flow-mediated dilation, **(E)** baseline pulse amplitude, **(F)** PAT ratio, **(G)** left ventricular ejection fraction, **(H)** left ventricular wall mass, **(I)** E/E’, **(J)** QTc duration, and **(K)** PR interval. Red squares indicate estimates in women, blue squares estimates in men. The model R^2^ is provided. Intima-media thickness, left ventricular wall mass, PR interval, E/E’, and baseline pulse amplitude were logarithmically transformed. Flow-mediated dilation was square-root transformed, model not adjusted for baseline diameter.

Multivariable-adjusted regression coefficients (betas) of classical CVD risk factors in relation to intermediate phenotypes are presented in Figure 1. Age, systolic blood pressure, and BMI were fairly consistently related to intermediate phenotypes in both sexes. Family history of myocardial infarction showed borderline statistical significance for a relation with IMT in women, but was otherwise not correlated with intermediate phenotypes. Whereas the direction of the relations in general was similar, the magnitude and significance of association showed sex-related differences. For IMT, diabetes did not reach a statistical significance in women. Systolic blood pressure was more strongly correlated with IMT in women (β = 0.03; 95% CI: 0.02–0.03; *P* < 0.001) compared to men (β = 0.01; 95% CI: 0.01–0.02; *P* < 0.001). In women, diabetes did not reach statistical significance in association with IMT compared to men. Sex interactions were observed for both age (*P* = 0.027) and systolic blood pressure (*P* = 0.033) and IMT.

Sex-specific differences were observed for age, diabetes, and systolic blood pressure in relation to ABI. Age only showed borderline significance in women, and diabetes did not reach statistical significance. In men, systolic blood pressure was not associated with ABI. A significant interaction was found between age and sex for FMD.

Baseline brachial artery diameter showed borderline significance for smoking in men, but not in women. In our sample, prevalent diabetes was negatively associated with baseline brachial artery diameter in men, whereas in women a positive correlation was observed. In contrast to men, systolic blood pressure did not reach statistical significance as a correlate of brachial artery diameter. For baseline brachial artery diameter, significant sex interactions were found for risk factors age (*P* = 0.0025), diabetes (*P* < 0.001), and BMI (*P* < 0.001).

Age was associated with FMD in women but not in men. Whereas systolic blood pressure was negatively associated with vascular function in males, it did not reach statistical significance in the female counterparts. BMI seemed to be more strongly associated with FMD in women (β = -0.07; 95% CI: -0.1 to 0.04; *P* < 0.001) versus men (β = -0.03; 95% CI: -0.05 to -0.02; *P* = 0.001). A significant interaction was found between age and sex for FMD. In a repeated regression, adjusted for CVRF and baseline brachial artery diameter, age was associated with FMD both in men and women. Systolic blood pressure was still negatively associated with FMD in men only; however, the association between BMI and endothelial function was no longer significant (β = 0.0; 95% CI: -0.02 to 0.03; *P* = 0.95 in women; β = 0.01; 95% CI: 0.00–0.03; *P* = 0.15 in men).

For baseline pulse amplitude, sex-specific differences were observed for diabetes, which did not reach statistical significance in men. Risk factors age (*P* < 0.001), diabetes (*P* = 0.0065), and dyslipidemia (*P* = 0.034) showed a significant interaction with sex for baseline pulse amplitude. The magnitude of association of systolic blood pressure was lower in women for PAT ratio compared to men [(β = 0.06; 95% CI: 0.05–0.08; *P* < 0.001) versus (β = 0.08; 95% CI: 0.07–0.1; *P* < 0.001)]. Dyslipidemia did not reach significance in men, but showed a clear negative association in women. Statistically significant were interactions between sex and variables age (*P* = 0.028), diabetes (*P* = 0.019), dyslipidemia (*P* = 0.0047), and BMI (*P* = 0.0027).

Left ventricular ejection fraction was strongly correlated with age in women, but not in men. Smoking status, diabetes, and BMI were significantly negatively associated with ejection fraction in men compared to women, where the point estimates of the beta coefficients were close to zero. The interaction between BMI and sex was statistically significant (*P* = 0.014). Current smoking, diabetes, and BMI were strongly related to left ventricular mass in women, but did not reach statistical significance in men. Only current smoking showed an interaction with sex (*P* = 0.0071). Except for dyslipidemia and a family history of myocardial infarction, risk factors were positively related to E/E’ in both sexes. Significant interactions resulted between sex and age (*P* = 0.017) as well as sex and BMI (*P* = 0.015). For ECG characteristics, differences by sex were observed for current smoking, which did not reach statistical significance in women as well as for dyslipidemia, which seemed to be positively related to PR interval. For QTc duration, interactions were significant between sex and risk factors: age (*P* < 0.001), systolic blood pressure (*P* < 0.001), and BMI (*P* = 0.0042).

The R^2^ values of the statistical models comprising CVD risk factors differed by sex. For IMT, baseline brachial artery diameter and baseline pulse amplitude, risk factors explained more of the variance of the phenotypes in women compared to men. For ABI and ECG variables, the R^2^ values were similar for both sexes. Overall, the amount of variance explained ranged from close to 0 for left ventricular ejection fraction in women to 43% of IMT variability in women.

**Figure 2:**
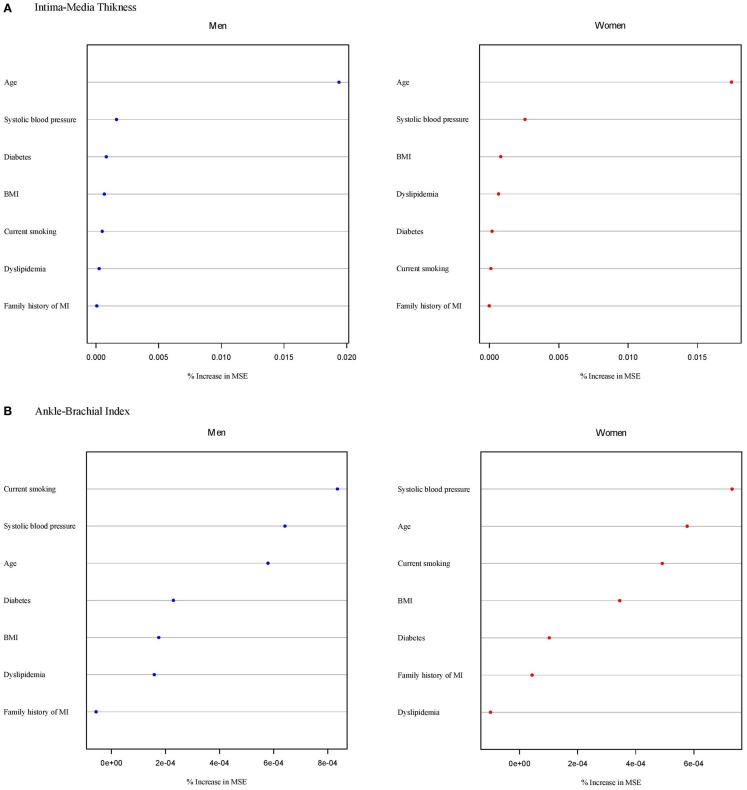

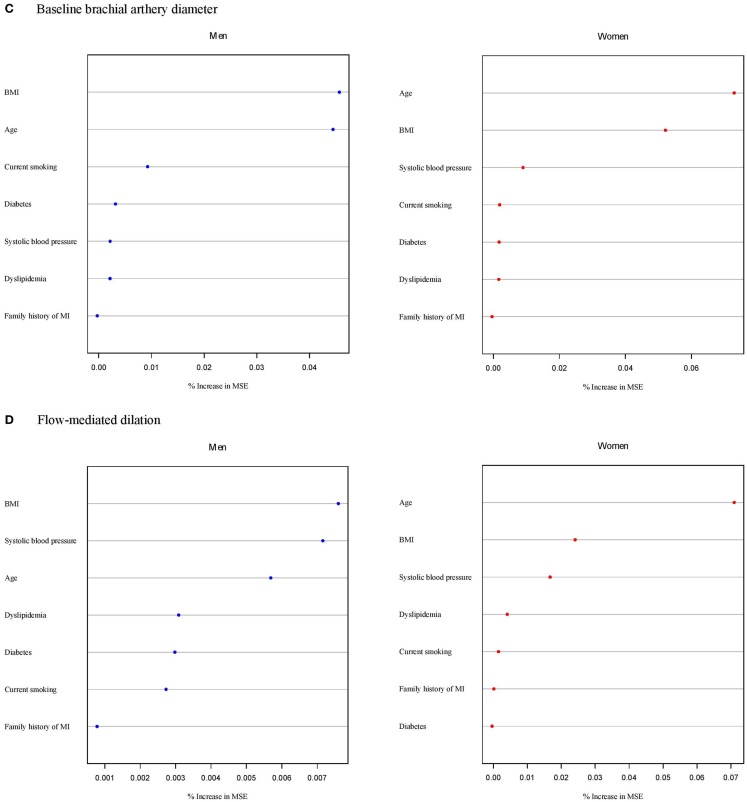

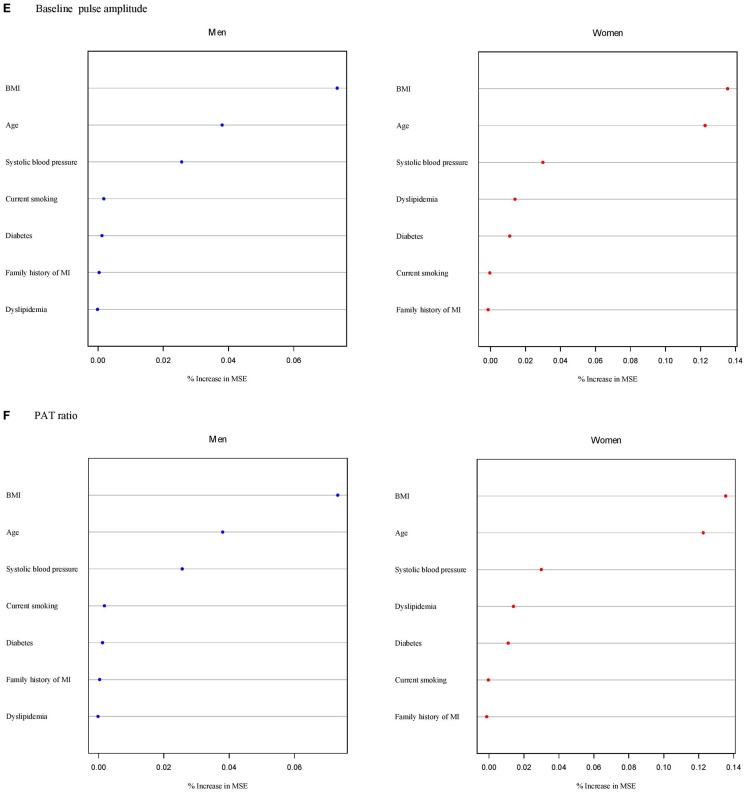

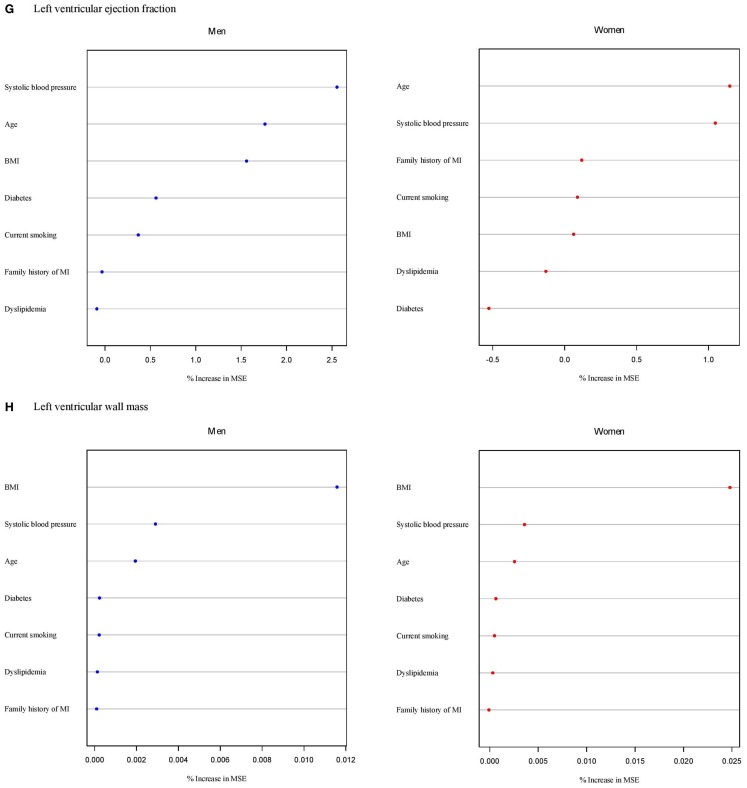

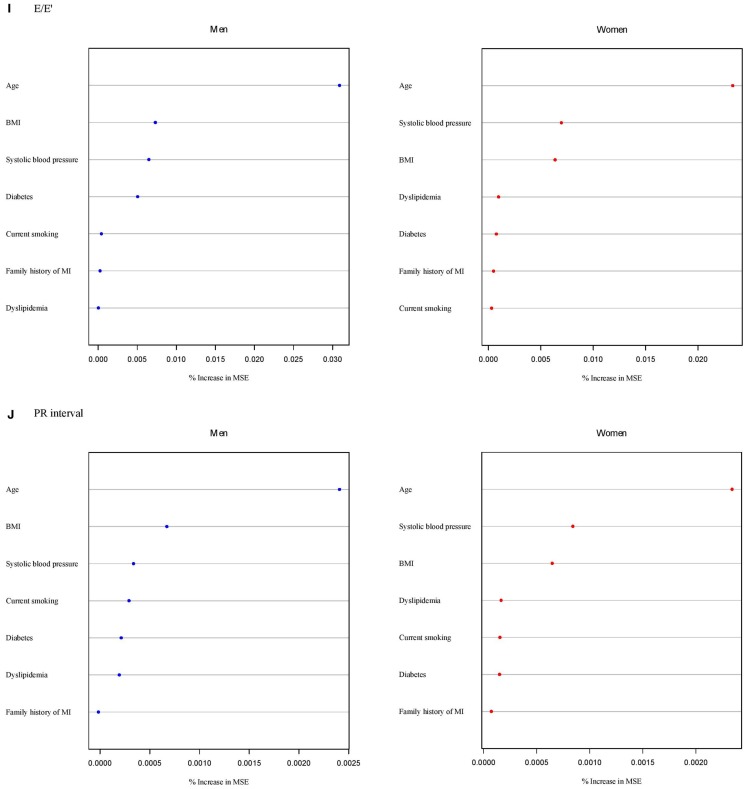

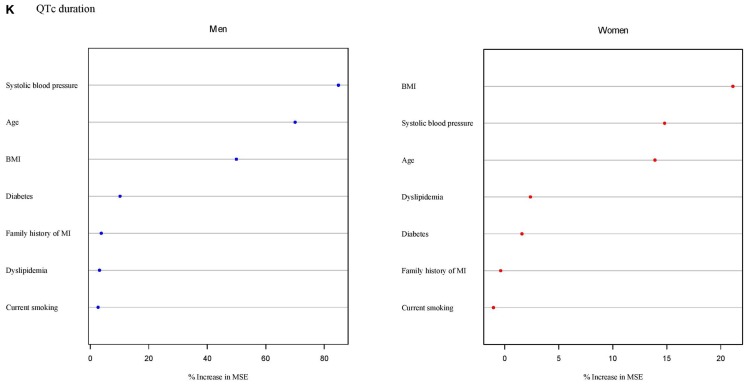
**Classical CVD risk factors in relation to intermediate phenotypes in order of importance from the most important (top) to less important variables (bottom) according to the random forest permutation variable importance measure stratified by sex**. Continuous variables of intermediate phenotypes are **(A)** intima-media thickness, **(B)** ankle-brachial index, **(C)** baseline brachial artery diameter, **(D)** flow-mediated dilation, **(E)** baseline pulse amplitude, **(F)** PAT ratio, **(G)** left ventricular ejection fraction, **(H)** left ventricular wall mass, **(I)** E/E’, **(J)** QTc duration, and **(K)** PR interval. MSE, mean squared error.

The results for the relative relevance of risk factors in relation to intermediate phenotypes assessed by random forest plots by sex are shown in Figure 2. Across phenotypes, age, systolic blood pressure, and BMI were the classical CVD risk factors consistently and most strongly related to the range of intermediate phenotypes. ABI additionally showed current smoking as one of the major predictors in both sexes. For IMT, diabetes seemed to be more important in men than in women.

### Intermediate phenotypes in relation to prevalent CVD

After adjustment for CVD risk factors, we observed moderate associations of intermediate phenotypes with CVD (Table 2). Differential associations were observed for IMT, which was related to coronary artery disease (OR 1.33; 95% CI: 1.1–1.62; *P* = 0.0031) and LEAD in men only (OR 1.3; 95% CI: 1.03–1.63; *P* = 0.025). Whereas ABI was correlated with all investigated CVD in men, it was only related to LEAD in women. Baseline brachial artery diameter correlated with stroke (OR 0.76; 95% CI: 0.58–0.99; *P* = 0.46) in men. Baseline pulse amplitude was associated with LEAD in women only (OR 0.65; 95% CI: 0.51–0.82; *P* < 0.001); PAT ratio was only statistically significantly correlated with heart failure in women (OR 0.76; 95% CI: 0.61–0.94; *P* = 0.010). Echocardiographic variables were associated with CVD in men. Left ventricular ejection fraction and E/E’ were related with heart failure in both sexes, whereas left ventricular wall mass associated with heart failure only in men (*P* = 0.0036). QTc was related with coronary artery disease and heart failure in both men and women. In our sample, no statistically significant association with CVD was detected for FMD (with or without adjustment for brachial artery diameter) and PR interval. Significant interactions were found solely between sex and heart failure for left ventricular ejection fraction (*P* = 0.013), for baseline brachial artery diameter (*P* = 0.029), and for PAT ratio (*P* = 0.016). Age-adjusted association analyses are presented in Table S1 in Supplementary Material.

**Table 2:** **Multivariable-adjusted odds ratios for intermediate phenotypes in relation to cardiovascular diseases**.

Variable	Men		Women	
	*N* = 2540		*N* = 2460	
	OR (95% CI)	*P* value	OR (95% CI)	*P* value
**Intima-media thickness**
Coronary artery disease	1.33 (1.1, 1.62)	0.0031	1.16 (0.82, 1.64)	0.39
Heart failure	1.00 (0.8, 1.24)	0.98	1.05 (0.83, 1.32)	0.70
Stroke	1.23 (0.91, 1.68)	0.18	1.02 (0.68, 1.52)	0.93
Myocardial infarction	1.19 (0.95, 1.49)	0.14	0.89 (0.59, 1.35)	0.60
LEAD	1.3 (1.03, 1.63)	0.025	1.18 (0.91, 1.53)	0.22
**Ankle-brachial index**
Coronary artery disease	0.68 (0.6, 0.77)	<0.001	0.79 (0.61, 1.02)	0.070
Heart failure	0.78 (0.68, 0.90)	<0.001	0.86 (0.72, 1.02)	0.090
Stroke	0.76 (0.64, 0.9)	0.0016	0.83 (0.62, 1.1)	0.19
Myocardial infarction	0.75 (0.65, 0.86)	<0.001	0.86 (0.63, 1.17)	0.32
LEAD	0.53 (0.46, 0.61)	<0.001	0.76 (0.64, 0.91)	0.0029
**Baseline brachial artery diameter**
Coronary artery disease	0.88 (0.74, 1.05)	0.16	0.85 (0.63, 1.16)	0.32
Heart failure^a^	0.87 (0.72, 1.06)	0.18	1.20 (0.98, 1.47)	0.082
Stroke	0.76 (0.58, 0.99)	0.046	0.88 (0.61, 1.26)	0.47
Myocardial infarction	0.96 (0.79, 1.17)	0.68	0.81 (0.56, 1.16)	0.25
LEAD	0.83 (0.67, 1.02)	0.079	1.03 (0.81, 1.29)	0.83
**Flow-mediated dilation**
Coronary artery disease	1.12 (0.95, 1.32)	0.19	1.1 (0.78, 1.55)	0.59
Heart failure	1.15 (0.95, 1.39)	0.16	1.02 (0.83, 1.27)	0.84
Stroke	1.26 (0.98, 1.64)	0.076	0.95 (0.65, 1.38)	0.77
Myocardial infarction	1.17 (0.97, 1.42)	0.10	0.97 (0.65, 1.45)	0.89
LEAD	0.98 (0.8, 1.2)	0.82	0.84 (0.67, 1.06)	0.14
**Baseline pulse amplitude**
Coronary artery disease	0.9 (0.75, 1.08)	0.27	1.05 (0.74, 1.49)	0.79
Heart failure	0.92 (0.74, 1.15)	0.46	1.16 (0.92, 1.46)	0.22
Stroke	0.9 (0.67, 1.22)	0.51	1.02 (0.69, 1.51)	0.92
Myocardial infarction	1.06 (0.84, 1.34)	0.62	1.34 (0.87, 2.06)	0.19
LEAD	0.85 (0.69, 1.07)	0.16	0.65 (0.51, 0.82)	<0.001
**Peripheral arterial tonometry**
Coronary artery disease	0.92 (0.76, 1.12)	0.40	0.96 (0.7, 1.31)	0.81
Heart failure^a^	1.11 (0.89, 1.37)	0.36	0.76 (0.61, 0.94)	0.010
Stroke	0.95 (0.7, 1.3)	0.75	0.89 (0.62, 1.27)	0.51
Myocardial infarction	0.78 (0.62, 0.99)	0.037	0.77 (0.53, 1.12)	0.17
LEAD	1.04 (0.83, 1.3)	0.75	1.24 (0.99, 1.57)	0.066
**Left ventricular ejection fraction**
Coronary artery disease	0.65 (0.57, 0.75)	<0.001	0.79 (0.62, 1.01)	0.057
Heart failure^a^	0.53 (0.45, 0.62)	<0.001	0.78 (0.65, 0.93)	0.0054
Stroke	0.84 (0.68, 1.03)	0.096	0.88 (0.65, 1.18)	0.38
Myocardial infarction	0.52 (0.45, 0.62)	<0.001	0.75 (0.57, 0.99)	0.044
LEAD	0.79 (0.67, 0.92)	0.0036	0.88 (0.72, 1.07)	0.20
**Left ventricular wall mass**
Coronary artery disease	1.33 (1.12, 1.57)	<0.001	1.29 (0.93, 1.79)	0.13
Heart failure	1.32 (1.10, 1.60)	0.0036	1.21 (0.98, 1.51)	0.081
Stroke	1.22 (0.94, 1.59)	0.14	1.08 (0.74, 1.57)	0.71
Myocardial infarction	1.6 (1.31, 1.94)	<0.001	1.54 (1.05, 2.27)	0.029
LEAD	1.09 (0.89, 1.34)	0.38	1.14 (0.89, 1.46)	0.29
**E/E′**
Coronary artery disease	1.21 (1.02, 1.44)	0.032	1.14 (0.85, 1.53)	0.37
Heart failure	1.54 (1.26, 1.88)	<0.001	1.53 (1.27, 1.85)	<0.001
Stroke	1.36 (1.03, 1.79)	0.03	0.95 (0.67, 1.35)	0.77
Myocardial infarction	1.42 (1.16, 1.73)	<0.001	1.64 (1.17, 2.3)	0.0041
LEAD	1.24 (1.01, 1.53)	0.04	0.92 (0.73, 1.15)	0.46
**PR interval**
Coronary artery disease	0.97 (0.82, 1.14)	0.70	1.17 (0.86, 1.59)	0.31
Heart failure	0.83 (0.68, 1.01)	0.061	1.09 (0.89, 1.32)	0.42
Stroke	1.12 (0.86, 1.46)	0.41	1.05 (0.74, 1.5)	0.79
Myocardial infarction	1.02 (0.84, 1.23)	0.88	0.97 (0.68, 1.38)	0.85
LEAD	0.85 (0.69, 1.04)	0.12	1.01 (0.81, 1.26)	0.94
**QTc duration**
Coronary artery disease	1.21 (1.04, 1.41)	0.015	1.56 (1.21, 2)	<0.001
Heart failure	1.49 (1.25, 1.77)	<0.001	1.44 (1.21, 1.71)	<0.001
Stroke	1.06 (0.83, 1.35)	0.66	1.34 (1, 1.8)	0.052
Myocardial infarction	1.16 (0.97, 1.38)	0.10	1.23 (0.9, 1.67)	0.19
LEAD	1.09 (0.9, 1.31)	0.40	1.14 (0.93, 1.39)	0.22

*Odds ratios (OR) per SD and 95% confidence intervals (CI) are presented. Multivariable-adjustment comprised age, body mass index, systolic blood pressure, current smoking, dyslipidemia, and a family history of MI. Intima-media thickness, left ventricular wall mass, PR interval, E/E’, and baseline pulse amplitude were log-transformed. Flow-mediated dilation was square-root transformed; presented model not adjusted for baseline diameter*.

*LEAD, lower extremity artery disease*.

*^a^Statistically significant sex interactions, *P* < 0.05*.

### Secondary analyses

In regression models, we did not observe significant interactions by sex. Associations did not change markedly, when regression analyses were adjusted for hypertension treatment or socioeconomic status (Tables S2A,B in Supplementary Material).

Associations in women younger than 51 years are presented in Table S3 in Supplementary Material. They largely resembled those in the total female sample. No statistically significant interaction by menopausal status was observed.

## Discussion

Contemporary data in our population-based cohort showed marked differences in a broad range of intermediate CVD phenotypes by sex. The direction of classical CVD risk factors with non-invasive CVD phenotypes was comparable in both sexes but often differed by magnitude and significance as clearly demonstrated for age, hypertension, and BMI. The ranking of risk factor correlates with intermediate phenotypes and CVD also revealed different patterns in men and women. Sex differences in the relation of intermediate phenotypes with prevalent CVD could not be fully explained by classical cardiovascular risk factors.

We observed an age-dependency of most intermediate phenotypes in both sexes, but no interaction by menopausal status. Prior examinations in the current sample had revealed rather linear than sudden changes in IMT and FMD in women after the age of menopause (10, 12). However, compared to women with a decrease in FMD, in men there was no significant association with age. This finding resulted in a statistically significant interaction by sex and may be due to the limited age range of the cohort. Changes in men may not be that profound for FMD in middle age compared to women. Across all other examined variables, a linear pattern toward less beneficial values of intermediate phenotypes could be demonstrated. These findings are in line with recent reports, stating that the effects of menopause are most likely less pronounced than formerly assumed (11, 23). For example, we have shown that circulating sex hormone concentrations were only modestly related to vascular phenotypes (24). However, the exact role of sex hormones and the genetic contribution to sex-specific cardiovascular aging remains to be determined (23, 25).

The fairly consistent correlation of hypertension and BMI with all vascular and cardiac phenotypes emphasizes the central importance of these risk factors for the development of early, possibly reversible stages of CVD in both, men and women.

In general, the relation of hypertension, smoking habits, overweight, and dyslipidemia to the intermediate phenotypes and manifest CVD could be confirmed. However, in our current results there appeared to be systematic differences in the magnitude of association of classical CVD risk factors with intermediate phenotypes. Several statistically significant interactions by sex could be demonstrated in multivariable regression models. Whereas differences in the distribution of classical CVD risk factors by sex are well accepted (2), we can demonstrate that there are likely differences in their impact on pathophysiological pathways leading to disease as reflected by intermediate phenotypes. The exact mechanisms remain to be elucidated. To account for possible sex-specific compliance with hypertension medication intake, we additionally adjusted for antihypertensive drugs. However, associations remained comparable after adjustment indicating that there may be other reasons for differential associations.

The relation of endophenotypes with manifest disease also differed by sex.

All intermediate phenotypes revealed differential associations with CVD by sex in multivariable-adjusted models. Whereas echocardiographic variables of systolic and diastolic function were associated with most CVD in men, statistically significant relations could only be observed for heart failure and myocardial infarction in women. Most striking sex-specific relations were demonstrated for ABI. This measure was associated with all CVD in men, but only with LEAD in women. IMT was associated with coronary artery disease and LEAD in men and only with stroke in women. Sex differences in associations of IMT and ABI with manifest atherosclerosis have been reported earlier (12, 13, 26). We can extend these findings toward a large population-based sample to five common CVDs. For baseline pulse amplitude, we could show an association with LEAD in women whereas in men, none of the associations reached statistical significance. Although our data cannot explain the exact mechanisms, such differential associations with CVD indicate possible sex differences not only in the characteristics of the intermediate phenotype, but also in the pathophysiological pathway toward disease manifestation. Since we are only at the beginning of describing such differences, future research needs to focus on mechanisms that link sex-specific intermediate cardiovascular function with hard disease endpoints and may help explain gender differences in cardiovascular disease outcomes.

We could further demonstrate that sex differences in intermediate phenotypes and prevalent CVD are not fully explained by classical cardiovascular risk factors alone. Even after accounting for socioeconomic status, differences in associations remained (27). Overall model variation explained by classical CVD risk factors was less than 50%. This finding indicates that there are other factors that are related to intermediate phenotypes and disease that may also help to understand sex differences. Additional factors, such as lifestyle habits beyond smoking, e.g. regular physical activity and a healthy diet may need to be considered to elucidate sex differences in susceptibility for CVD. Women tend to have a healthier lifestyle, although overall full adherence to healthy lifestyle recommendations still needs improvement (2, 28). Furthermore, additional risk factors need to be identified that may help to explain differences in intermediate phenotypes and CVD.

We cannot derive specific treatment recommendations from our current observational data. However, we know that classical CVD risk factors are modifiable. Treatment of dyslipidemia, hypertension, and smoking cessation decrease mortality rates of coronary artery disease (29). Since there are differences in the prevalence of cardiovascular disease risk factors by sex and we show different associations with intermediate phenotypes, it can be assumed that sex-specific interventions may be needed. Awareness of disease risk factors in women has improved over the last decades (30). Programs, such as those implemented in the United States, which specifically address CVD risk factors and early disease in women will help to resolve sex disparities in CVD. Sex-specific and individualized risk factor treatment and prevention, as recommended by recent guidelines, will enhance cardiovascular health in women and men (31).

### Limitations

Our cross-sectional data rely on the association of intermediate phenotypes with a comparatively small number of prevalent CVD cases, which may be affected by reverse causation in prevalent disease and other differences related to the diagnosis, treatment, and coping with the disease. Sex-differences in atherosclerosis have been reported to be most pronounced in the coronary vascular bed which was not sufficiently reflected in our study (26). At the population level, we cannot provide in depth mechanistic insights, but our results need to be viewed as hypothesis-generating for experimental follow-up. Strengths of our study are the broad range of high-quality intermediate cardiovascular phenotypes and CVD risk factor assessment in a sample, which is representative of the middle-aged general population.

In conclusion, we observed clear sex differences in a large number of non-invasive intermediate phenotypes of cardiovascular structure and function, which was partially explained by differential classical risk factor associations. Age, BMI, and hypertension showed most relevant differences. Sex-specific relations of intermediate phenotypes with prevalent disease were not fully explained by differential distribution of classical CVD risk factors. Our data may contribute to the understanding of sex differences in the susceptibility to CVD, early stages, and manifestation of disease. Such knowledge underscores the necessity of sex-specific CVD risk assessment and may help to better tailor future preventive efforts in the general population.

## Conflict of Interest Statement

The authors declare that the research was conducted in the absence of any commercial or financial relationships that could be construed as a potential conflict of interest.

## Supplementary Material

The Supplementary Material for this article can be found online at http://journal.frontiersin.org/article/10.3389/fcvm.2015.00015

## Abbreviations

ABI, ankle-brachial index; BMI, body mass index; CI, confidence interval; CVD, cardiovascular disease; ECG, electrocardiogram; IMT, intima-media thickness; LEAD, lower extremity artery disease; MI, myocardial infarction; MSE, mean squared error; OR, odds ratio
