# Supplementary material for: Sex Differences in Correlates of Intermediate Phenotypes and Prevalent Cardiovascular Disease in the General Population
Source: Front Cardiovasc Med. 2015 Apr 15;2:15. doi: 10.3389/fcvm.2015.00015 (PMC4671364; doi:10.3389/fcvm.2015.00015)
Supplement: Supplementary file 1 [file Data_Sheet_1.PDF]

Supplementary Material

Sex Differences in Correlates of Hypertension-Related Intermediate Phenotypes and Prevalent Cardiovascular Disease in the General Population

Renate B. Schnabel, MD, MSc; Philipp. S. Wild, MD, MSc; Jürgen Prochaska, MD; Francisco M. Ojeda, PhD; Tanja Zeller, PhD; Nargiz Rzayeva, MSc; Ariana Ebrahim; Karl J. Lackner, MD; Manfred E. Beutel, MD; Norbert Pfeiffer; MD; Christoph R. Sinning, MD; Sabine Oertelt-Prigione, MD; Vera Regitz-Zagrosek, MD; Harald Binder, PhD; Thomas Münzel, MD; Stefan Blankenberg, MD for the Gutenberg Health Study investigators

Supplementary Methods

Statistical methods

Random forest analyses

To understand the relative importance of classical CVD risk factors in relation to intermediate phenotypes by sex, a random forest was calculated for each cardiovascular phenotype[1] with the risk factors as predictors. The permutation variable importance measure of the forest was computed and used to rank the predictors. A random forest consists of a large number of classification and regression trees which is grown via bootstrap resampling. The individuals that are not used when growing a particular tree, the so called out-of-bag sample, can be used to assess the predictive accuracy of that tree. The permutation variable importance measured is computed as follows, first, the predictive accuracy of each tree is computed using the corresponding out-bag-sample, then the values of a given predictor are shuffled on the out-bag-sample and the predictive accuracy of the tree is computed again on this permuted sample. The decrease in accuracy is averaged over the forest and this is the permutation importance measure. The idea behind this measure is that if a variable is not important, the predictive accuracy after permuting its values will not be that different from the predictive accuracy using the original values. The accuracy measure used was the mean squared error [MSE]. In our analyses we produced 2500 trees per forest.

Supplementary Results

Supplementary Figure 1. Distribution of intermediate phenotypes by sex. Blue boxplots are for men, red boxplots for women.

Supplementary Figure 2. Distribution curves of intermediate phenotypes by age decade using quantile regression. Age is modeled using degree 2 B-splines. The lines indicate the 25th, 50th, and 75th percentile.

Supplementary Tables

Supplementary Table 1. Age-adjusted odds ratios for intermediate phenotypes in relation to cardiovascular diseases.

Variable	Men N=2540		Women N=2460	
	**OR (95%-CI)**	**P Value**	**OR (95%-CI)**	**P Value**
**Mean intima-media thickness**				
Coronary artery disease	1.36 (1.13,1.63)	0.0013	1.16 (0.84,1.61)	0.36
Heart failure	1.09 (0.88,1.35)	0.42	1.13 (0.92,1.40)	0.25
Stroke	1.22 (0.9,1.65)	0.21	1.03 (0.7,1.54)	0.87
Myocardial infarction	1.24 (0.99,1.54)	0.057	0.91 (0.61,1.34)	0.62
LEAD	1.42 (1.14,1.78)	0.0020	1.18 (0.92,1.52)	0.18
**Ankle-brachial index**				
Coronary artery disease	0.67 (0.6,0.76)	<0.001	0.8 (0.63,1)	0.050
Heart failure	0.76 (0.66,0.86)	<0.001	0.91 (0.77,1.07)	0.25
Stroke	0.77 (0.65,0.91)	0.0019	0.83 (0.63,1.1)	0.19
Myocardial infarction	0.72 (0.64,0.82)	<0.001	0.86 (0.64,1.14)	0.29
LEAD*	0.51 (0.44,0.59)	<0.001	0.78 (0.66,0.93)	0.0061
**Baseline brachial artery diameter**				
Coronary artery disease	0.9 (0.77,1.06)	0.22	0.98 (0.73,1.31)	0.88
Heart failure *	0.99 (0.82,1.19)	0.91	1.44 (1.18,1.74)	<0.001
Stroke	0.8 (0.61,1.04)	0.089	0.92 (0.65,1.3)	0.63
Myocardial infarction	0.96 (0.79,1.16)	0.64	0.97 (0.68,1.37)	0.85
LEAD	0.87 (0.71,1.06)	0.15	1.15 (0.92,1.43)	0.22
**Flow-mediated dilation**				
Coronary artery disease	1.11 (0.95,1.31)	0.19	1.07 (0.76,1.5)	0.69
Heart failure	1.06 (0.88,1.28)	0.53	0.92 (0.75,1.14)	0.45
Stroke	1.26 (0.98,1.63)	0.070	0.94 (0.65,1.37)	0.76
Myocardial infarction	1.16 (0.97,1.4)	0.11	0.91 (0.62,1.35)	0.65
LEAD	0.93 (0.76,1.13)	0.46	0.8 (0.64,1.01)	0.056
**Baseline pulse amplitude**				
Coronary artery disease	0.99 (0.83,1.18)	0.93	1.26 (0.9,1.75)	0.18
Heart failure	1.07 (0.87,1.32)	0.51	1.47 (1.18,1.83)	<0.001
Stroke	0.98 (0.73,1.31)	0.87	1.09 (0.76,1.58)	0.55
Myocardial infarction	1.16 (0.94,1.45)	0.17	1.72 (1.13,2.61)	0.011
LEAD	0.93 (0.76,1.14)	0.48	0.76 (0.61,0.95)	0.017
**PAT ratio**				
Coronary artery disease	0.79 (0.66,0.94)	0.0092	0.77 (0.58,1.04)	0.086
Heart failure*	0.9 (0.73,1.09)	0.28	0.65 (0.53,0.79)	<0.001
Stroke	0.84 (0.63,1.13)	0.26	0.83 (0.59,1.16)	0.27

Variable	Men N=2540		Women N=2460	
	**OR (95%-CI)**	**P Value**	**OR (95%-CI)**	**P Value**
Myocardial infarction	0.65 (0.52,0.81)	<0.001	0.61 (0.43,0.86)	0.0053
LEAD	0.93 (0.76,1.14)	0.49	1.1 (0.88,1.36)	0.40
**Left ventricular ejection fraction**				
Coronary artery disease	0.64 (0.56,0.73)	<0.001	0.75 (0.59,0.96)	0.019
Heart failure*	0.51 (0.44,0.60)	<0.001	0.80 (0.67,0.95)	0.010
Stroke	0.81 (0.66,0.99)	0.041	0.86 (0.64,1.16)	0.32
Myocardial infarction	0.51 (0.44,0.6)	<0.001	0.73 (0.56,0.96)	0.025
LEAD	0.75 (0.64,0.88)	<0.001	0.88 (0.72,1.06)	0.18
**Left ventricular wall mass**				
Coronary artery disease	1.35 (1.16,1.58)	<0.001	1.51 (1.15,2)	0.0034
Heart failure	1.54 (1.29,1.83)	<0.001	1.70 (1.41,2.05)	<0.001
Stroke	1.27 (0.99,1.63)	0.057	1.12 (0.8,1.56)	0.50
Myocardial infarction	1.61 (1.34,1.93)	<0.001	1.77 (1.27,2.45)	<0.001
LEAD	1.19 (0.99,1.43)	0.071	1.3 (1.05,1.61)	0.014
**E/E‘**				
Coronary artery disease	1.25 (1.06,1.47)	0.0081	1.23 (0.94,1.62)	0.13
Heart failure	1.70 (1.41,2.06)	<0.001	1.75 (1.47,2.09)	<0.001
Stroke	1.38 (1.06,1.8)	0.016	0.97 (0.69,1.37)	0.88
Myocardial infarction	1.5 (1.24,1.81)	<0.001	1.7 (1.24,2.33)	<0.001
LEAD*	1.4 (1.14,1.7)	0.001	1 (0.8,1.24)	0.97
**PR interval**				
Coronary artery disease	1.03 (0.88,1.21)	0.73	1.29 (0.96,1.73)	0.091
Heart failure	0.92 (0.76,1.10)	0.36	1.17 (0.97,1.40)	0.10
Stroke	1.17 (0.91,1.52)	0.22	1.09 (0.77,1.54)	0.65
Myocardial infarction	1.08 (0.89,1.3)	0.44	1.09 (0.77,1.55)	0.63
LEAD	0.89 (0.73,1.09)	0.25	1.06 (0.85,1.32)	0.61
**QTc duration**				
Coronary artery disease*	1.19 (1.03,1.38)	0.019	1.55 (1.23,1.95)	<0.001
Heart failure	1.59 (1.35,1.88)	<0.001	1.63 (1.39,1.93)	<0.001
Stroke	1.07 (0.85,1.37)	0.56	1.32 (0.99,1.76)	0.055
Myocardial infarction	1.17 (0.98,1.39)	0.079	1.31 (0.99,1.73)	0.062
LEAD	1.15 (0.96,1.38)	0.13	1.18 (0.97,1.44)	0.10

Odds ratios [OR] per standard deviation and 95% confidence intervals [CI] are presented. All models are adjusted for age. Intima-media thickness, left ventricular wall mass, PR interval, E/E’ and baseline pulse amplitude were log-transformed. Flow-mediated dilation was square-root transformed, presented model not adjusted for baseline diameter Heart failure definition comprised left ventricular ejection fraction.* Statistically significant sex interactions.

Supplementary Table 2. Multivariable linear regressions for intermediate phenotypes adjusted for classical CVD risk factors and additionally for a) hypertension medication, and b) socioeconomic status.

**Supplementary Table 2a.**	**Men N=2540**		**Women N=2460**	
**Variable**	**Beta (95%-CI)**	**P Value**	**Beta (95%-CI)**	**P Value**
**Intima-media thickness**				
Age*	0.11 (0.10,0.11)	<0.001	0.09 (0.09,0.10)	<0.001
Current smoking	0.01 (0.01,0.02)	<0.001	0.01 (0.01,0.02)	<0.001
Diabetes*	0.01 (0.0,0.02)	0.0071	0.0 (-0.01,0.0)	0.72
Systolic blood pressure	0.01 (0.01,0.02)	<0.001	0.02 (0.02,0.03)	<0.001
Family history of MI	0.0 (0.0,0.01)	0.17	0.01 (0.0,0.01)	0.052
Dyslipidemia	0.01 (0.0,0.02)	0.0063	0.01 (0.01,0.02)	<0.001
BMI	0.01 (0.01,0.02)	<0.001	0.01 (0.0,0.02)	0.0012
Antihypertensive medication	0.01 (0.0,0.01)	0.17	0.01 (0.01,0.02)	<0.001
**Ankle-brachial index**				
Age	0.0 (-0.01,0.0)	0.36	0.0 (-0.01,0.0)	0.20
Current smoking	-0.02 (-0.02,-0.01)	<0.001	-0.01 (-0.01,-0.01)	<0.001
Diabetes*	-0.01 (-0.02,0.0)	0.0010	0.0 (0.0,0.0)	0.83
Systolic blood pressure	0.0 (-0.01,0.0)	0.36	-0.01 (-0.01,0.0)	0.0028
Family history of MI	0.0 (-0.01,0.01)	0.87	0.0 (-0.01,0.0)	0.17
Dyslipidemia	0.0 (-0.01,0.0)	0.36	0.0 (-0.01,0.0)	0.57
BMI	0.01 (0.0,0.02)	<0.001	0.01 (0.0,0.01)	<0.001
Antihypertensive medication*	-0.02 (-0.02,-0.01)	<0.001	-0.01 (-0.01,0)	0.0021
**Baseline brachial artery diameter**				
Age*	0.16 (0.13,0.18)	<0.001	0.20 (0.17,0.22)	<0.001
Current smoking	-0.02 (-0.05,0.0)	0.041	0.0 (-0.02,0.02)	0.76
Diabetes*	-0.03 (-0.05,-0.01)	0.017	0.03 (0.01,0.05)	0.0062
Systolic blood pressure	0.04 (0.02,0.07)	0.001	0.01 (-0.01,0.04)	0.27
Family history of MI	0.0 (-0.02,0.03)	0.79	0.0 (-0.02,0.02)	0.92
Dyslipidemia	-0.01 (-0.03,0.01)	0.49	-0.01 (-0.03,0.02)	0.57
BMI*	0.17 (0.15,0.20)	<0.001	0.15 (0.13,0.17)	<0.001
Antihypertensive medication*	-0.01 (-0.04,0.01)	0.36	0.03 (0.0,0.05)	0.019
**Flow-mediated dilation**				
Age*	0.02 (0.0,0.04)	0.046	-0.16 (-0.19,-0.14)	<0.001
Current smoking	0.02 (0.0,0.03)	0.094	-0.01 (-0.03,0.02)	0.58
Diabetes	-0.02 (-0.04,0.0)	0.064	-0.02 (-0.04,0.01)	0.21
Systolic blood pressure	-0.03 (-0.05,-0.02)	<0.001	-0.02 (-0.05,0.01)	0.21

Supplementary Table 2a.	Men N=2540		Women N=2460	
**Variable**	**Beta (95%-CI)**	**P Value**	**Beta (95%-CI)**	**P Value**
Family history of MI	0.01 (-0.01,0.03)	0.32	0.0 (-0.03,0.02)	0.79
Dyslipidemia	0.0 (-0.02,0.02)	0.80	0.0 (-0.03,0.02)	0.97
BMI	-0.03 (-0.05,-0.01)	<0.001	-0.06 (-0.08,-0.03)	<0.001
Antihypertensive medication*	-0.02 (-0.04,0.0)	0.10	-0.05 (-0.08,-0.02)	<0.001
**Baseline pulse amplitude**				
Age*	0.13 (0.09,0.16)	<0.001	0.23 (0.19,0.27)	<0.001
Current smoking	0.03 (0,0.06)	0.035	0.05 (0.02,0.09)	0.0027
Diabetes*	0 (-0.03,0.03)	0.98	0.05 (0.01,0.08)	0.0063
Systolic blood pressure	-0.07 (-0.1,-0.04)	<0.001	-0.07 (-0.11,-0.03)	<0.001
Family history of MI	0 (-0.03,0.03)	0.9	-0.02 (-0.05,0.01)	0.24
Dyslipidemia*	0.04 (0.01,0.06)	0.016	0.07 (0.04,0.11)	<0.001
BMI	0.2 (0.17,0.23)	<0.001	0.25 (0.21,0.29)	<0.001
Antihypertensive medication*	-0.03 (-0.06,0.01)	0.11	0.03 (-0.01,0.07)	0.10
**PAT ratio**				
Age	-0.06 (-0.07,-0.04)	<0.001	-0.03 (-0.05,-0.01)	<0.001
Current smoking	-0.02 (-0.04,-0.01)	0.0023	-0.02 (-0.03,0)	0.053
Diabetes*	-0.01 (-0.02,0.01)	0.40	-0.03 (-0.04,-0.01)	<0.001
Systolic blood pressure	0.08 (0.07,0.1)	<0.001	0.07 (0.05,0.09)	<0.001
Family history of MI	-0.01 (-0.02,0.01)	0.31	0.01 (0,0.03)	0.16
Dyslipidemia*	0 (-0.02,0.01)	0. 52	-0.03 (-0.05,-0.02)	<0.01
BMI*	-0.1 (-0.12,-0.09)	<0.001	-0.09 (-0.1,-0.07)	<0.001
Antihypertensive medication	-0.02 (-0.04,-0.01)	0.0092	-0.03 (-0.04,-0.01)	0.0054
**Left ventricular ejection fraction**				
Age	0.11 (-0.21,0.44)	0.50	0.32 (0.03,0.6)	0.028
Current smoking	-0.33 (-0.62,-0.05)	0.021	-0.22 (-0.46,0.02)	0.078
Diabetes	-0.27 (-0.56,0.03)	0.075	-0.04 (-0.29,0.2)	0.73
Systolic blood pressure	0.25 (-0.05,0.55)	0.097	-0.11 (-0.39,0.16)	0.41
Family history of MI	-0.14 (-0.42,0.14)	0.33	-0.08 (-0.31,0.16)	0.52
Dyslipidemia	0.08 (-0.2,0.36)	0.58	0 (-0.25,0.24)	0.97
BMI	-0.4 (-0.7,-0.11)	0.0078	-0.07 (-0.32,0.19)	0.60
Antihypertensive medication*	-0.37 (-0.69,-0.05)	0.025	0.15 (-0.11,0.42)	0.26
**Left ventricular wall mass**				
Age	0.02 (0.01,0.03)	0.0024	0.02 (0.01,0.03)	<0.001
Current smoking*	0 (-0.01,0)	0.35	0.01 (0,0.02)	0.0078

Supplementary Table 2a.	Men N=2540		Women N=2460	
**Variable**	**Beta (95%-CI)**	**P Value**	**Beta (95%-CI)**	**P Value**
Diabetes	0 (-0.01,0.01)	0.64	0.01 (0,0.02)	0.049
Systolic blood pressure	0.02 (0.01,0.03)	<0.001	0.03 (0.02,0.04)	<0.001
Family history of MI	0 (-0.01,0.01)	0.44	0 (-0.01,0.01)	0.86
Dyslipidemia	0 (-0.01,0.01)	0.81	0 (-0.01,0.01)	0.71
BMI*	0.08 (0.07,0.09)	<0.001	0.11 (0.1,0.12)	<0.001
Antihypertensive medication	0.02 (0.01,0.03)	<0.0001	0.02 (0.01,0.03)	<0.001
**E/E'**				
Age*	0.12 (0.1,0.13)	<0.001	0.1 (0.09,0.11)	<0.001
Current smoking	0.02 (0.01,0.03)	<0.001	0.02 (0.01,0.03)	<0.001
Diabetes	0.03 (0.02,0.04)	<0.001	0.01 (0,0.02)	0.043
Systolic blood pressure	0.03 (0.02,0.04)	<0.001	0.03 (0.02,0.05)	<0.001
Family history of MI	0 (-0.01,0.01)	0.86	0.01 (0,0.02)	0.14
Dyslipidemia	0 (-0.01,0.02)	0.41	0 (-0.01,0.01)	0.48
BMI*	0.05 (0.04,0.06)	<0.001	0.04 (0.03,0.05)	<0.001
Antihypertensive medication	0.03 (0.02,0.04)	<0.001	0.04 (0.02,0.05)	<0.001
**PR interval**				
Age	0.03 (0.02,0.04)	<0.001	0.03 (0.02,0.04)	<0.001
Current smoking	-0.01 (-0.02,-0.01)	<0.001	-0.01 (-0.01,0)	0.054
Diabetes	0 (-0.01,0)	0.54	-0.01 (-0.01,0)	0.063
Systolic blood pressure	-0.01 (-0.01,0)	0.0062	-0.01 (-0.02,0)	0.0010
Family history of MI	0 (-0.01,0)	0.61	0 (-0.01,0)	0.49
Dyslipidemia	0 (0,0.01)	0.26	0.01 (0,0.01)	0.015
BMI	0.02 (0.01,0.02)	<0.001	0.02 (0.01,0.02)	<0.001
Antihypertensive medication	0.01 (0,0.02)	0.0027	0.01 (0.01,0.02)	<0.001
**QTc Duration**				
Age*	5.19 (4.11,6.27)	<0.001	2.01 (0.98,3.04)	<0.001
Current smoking	-0.21 (-1.15,0.73)	0.66	-1.4 (-2.27,-0.52)	0.0017
Diabetes	1.29 (0.31,2.27)	0.010	1.07 (0.18,1.96)	0.019
Systolic blood pressure*	4.81 (3.83,5.8)	<0.001	2.27 (1.29,3.25)	<0.001
Family history of MI	-0.74 (-1.67,0.18)	0.12	-0.76 (-1.62,0.09)	0.081
Dyslipidemia	0.63 (-0.31,1.57)	0.19	-0.38 (-1.26,0.5)	0.40
BMI*	4.3 (3.31,5.29)	<0.001	3.35 (2.42,4.28)	<0.001
Antihypertensive medication	0.38 (-0.69,1.46)	0.49	0.36 (-0.61,1.32)	0.47

Supplementary Table 2b.	Men N=2540		Women N=2460	
**Variable**	**Beta (95%-CI)**	**P Value**	**Beta (95%-CI)**	**P Value**
**Intima-media thickness**				
Age	0.10 (0.1,0.11)	<0.001	0.1 (0.09,0.1)	<0.001
Current smoking	0.01 (0.01,0.02)	<0.001	0.01 (0.01,0.02)	<0.001
Diabetes	0.01 (0.0,0.01)	0.012	0 (-0.01,0.01)	0.88
Systolic blood pressure	0.01 (0.01,0.02)	<0.001	0.02 (0.02,0.03)	<0.001
Family history of MI	0 (0,0.01)	0.13	0.01 (0,0.01)	0.061
Dyslipidemia	0.01 (0,0.02)	0.0070	0.01 (0.01,0.02)	<0.001
BMI	0.01 (0,0.02)	0.0016	0.01 (0.01,0.02)	<0.001
Socioeconomic status*	-0.01 (-0.02,-0.01)	<0.001	0 (-0.01,0)	0.19
**Ankle-brachial index**				
Age	-0.01 (-0.01,0)	0.033	0 (-0.01,0)	0.12
Current smoking	-0.01 (-0.02,-0.01)	<0.001	-0.01 (-0.01,-0.01)	<0.001
Diabetes*	-0.01 (-0.02,-0.01)	<0.001	0 (-0.01,0)	0.64
Systolic blood pressure	0 (-0.01,0)	0.35	-0.01 (-0.01,0)	0.0019
Family history of MI	0 (-0.01,0.01)	0.88	0 (-0.01,0)	0.12
Dyslipidemia	0 (-0.01,0)	0.25	0 (-0.01,0)	0.47
BMI	0.01 (0,0.02)	0.0017	0.01 (0,0.01)	<0.001
Socioeconomic status	0.01 (0.01,0.02)	<0.001	0.01 (0,0.01)	0.017
**Baseline brachial artery diameter**				
Age*	0.15 (0.12,0.17)	<0.001	0.2 (0.18,0.23)	<0.001
Current smoking*	-0.03 (-0.05,0)	0.018	0.01 (-0.02,0.03)	0.64
Diabetes*	-0.03 (-0.06,-0.01)	0.0045	0.03 (0.01,0.05)	0.0043
Systolic blood pressure	0.04 (0.02,0.06)	0.0013	0.02 (-0.01,0.04)	0.19
Family history of MI	0 (-0.02,0.03)	0.78	0 (-0.02,0.02)	0.93
Dyslipidemia	-0.01 (-0.03,0.02)	0.54	-0.01 (-0.03,0.02)	0.62
BMI*	0.17 (0.14,0.19)	<0.001	0.16 (0.13,0.18)	<0.001
Socioeconomic status	-0.03 (-0.05,0)	0.024	-0.01 (-0.03,0.01)	0.45
**Flow-mediated dilation**				
Age*	0.01 (-0.01,0.03)	0.36	-0.18 (-0.21,-0.15)	<0.001
Current smoking	0.01 (0,0.03)	0.13	-0.01 (-0.03,0.02)	0.51
Diabetes	-0.02 (-0.04,0)	0.031	-0.02 (-0.04,0.01)	0.15
Systolic blood pressure	-0.03 (-0.05,-0.02)	<0.001	-0.02 (-0.05,0)	0.10
Family history of MI	0.01 (-0.01,0.03)	0.27	-0.01 (-0.03,0.02)	0.58
Dyslipidemia	0 (-0.02,0.01)	0.70	0 (-0.03,0.02)	0.91
BMI	-0.04 (-0.05,-0.02)	<0.001	-0.07 (-0.1,-0.04)	<0.001

Supplementary Table 2b.	Men N=2540		Women N=2460	
**Variable**	**Beta (95%-CI)**	**P Value**	**Beta (95%-CI)**	**P Value**
Socioeconomic status	-0.01 (-0.03,0.01)	0.15	0 (-0.02,0.03)	0.92
**Baseline pulse amplitude**				
Age*	0.13 (0.1,0.16)	<0.001	0.24 (0.2,0.28)	<0.001
Current smoking	0.04 (0.01,0.06)	0.015	0.05 (0.02,0.09)	0.0029
Diabetes*	0 (-0.03,0.03)	0.83	0.05 (0.02,0.09)	0.0041
Systolic blood pressure	-0.07 (-0.1,-0.04)	<0.001	-0.07 (-0.11,-0.03)	<0.001
Family history of MI	0 (-0.03,0.02)	0.73	-0.02 (-0.05,0.03)	0.32
Dyslipidemia*	0.03 (0.01,0.06)	0.02	0.08 (0.04,0.11)	<0.001
BMI	0.2 (0.17,0.23)	<0.001	0.26 (0.22,0.29)	<0.001
Socioeconomic status	0.04 (0.01,0.07)	0.0032	0.01 (-0.03,0.05)	0.60
**PAT ratio**				
Age*	-0.07 (-0.08,-0.05)	<0.001	-0.04 (-0.06,-0.02)	<0.001
Current smoking	-0.02 (-0.04,-0.01)	0.0039	-0.02 (-0.03,0)	0.058
Diabetes*	-0.01 (-0.02,0.01)	0.22	-0.03 (-0.05,-0.01)	<0.001
Systolic blood pressure	0.08 (0.07,0.1)	<0.001	0.06 (0.05,0.08)	<0.001
Family history of MI	-0.01 (-0.02,0.01)	0.28	0.01 (-0.01,0.03)	0.24
Dyslipidemia*	-0.01 (-0.02,0.01)	0.28	-0.03 (-0.05,-0.02)	<0.001
BMI*	-0.1 (-0.12,-0.09)	<0.001	-0.09 (-0.11,-0.08)	<0.001
Socioeconomic status	0 (-0.01,0.02)	0.77	0 (-0.02,0.01)	0.62
**Left ventricular ejection fraction**				
Age	0.03 (-0.27,0.34)	0.83	0.36 (0.08,0.64)	0.013
Current smoking	-0.28 (-0.57,0)	0.052	-0.21 (-0.45,0.03)	0.093
Diabetes	-0.32 (-0.61,-0.03)	0.030	0.01 (-0.23,0.25)	0.93
Systolic blood pressure	0.27 (-0.03,0.56)	0.079	-0.11 (-0.38,0.16)	0.43
Family history of MI	-0.17 (-0.45,0.1)	0.22	-0.08 (-0.32,0.16)	0.51
Dyslipidemia	0.04 (-0.24,0.32)	0.80	0 (-0.24,0.25)	0.98
BMI*	-0.4 (-0.69,-0.1)	0.0087	0.02 (-0.24,0.27)	0.89
Socioeconomic status	0.41 (0.12,0.7)	0.0057	0.05 (-0.21,0.3)	0.71
**Left ventricular wall mass**				
Age	0.02 (0.01,0.03)	<0.001	0.03 (0.02,0.04)	<0.001
Current smoking*	0 (-0.01,0)	0.29	0.01 (0,0.02)	0.0057
Diabetes	0.01 (0,0.01)	0.28	0.01 (0,0.02)	0.014
Systolic blood pressure	0.02 (0.01,0.03)	<0.001	0.03 (0.02,0.04)	<0.001
Family history of MI	0 (0,0.01)	0.39	0 (-0.01,0.01)	0.80
Dyslipidemia	0 (-0.01,0.01)	0.85	0 (-0.01,0.01)	0.83

Supplementary Table 2b.	Men N=2540		Women N=2460	
**Variable**	**Beta (95%-CI)**	**P Value**	**Beta (95%-CI)**	**P Value**
BMI*	0.08 (0.07,0.09)	<0.001	0.12 (0.11,0.13)	<0.001
Socioeconomic status	0 (-0.01,0.01)	0.75	0 (-0.01,0.01)	0.42
**E/E’**				
Age*	0.12 (0.11,0.14)	<0.001	0.11 (0.09,0.12)	<0.001
Current smoking	0.02 (0.01,0.03)	0.0014	0.02 (0.01,0.03)	<0.001
Diabetes	0.03 (0.02,0.05)	<0.001	0.01 (0,0.02)	0.017
Systolic blood pressure	0.03 (0.02,0.05)	<0.001	0.04 (0.03,0.05)	<0.001
Family history of MI	0 (-0.01,0.01)	0.66	0.01 (0,0.02)	0.087
Dyslipidemia	0.01 (-0.01,0.02)	0.35	0.01 (-0.01,0.02)	0.31
BMI*	0.05 (0.04,0.06)	<0.001	0.04 (0.03,0.05)	<0.001
Socioeconomic status	-0.02 (-0.03,-0.01)	0.0014	-0.01 (-0.02,0)	0.27
**PR interval**				
Age	0.03 (0.03,0.04)	<0.001	0.04 (0.03,0.04)	<0.001
Current smoking	-0.01 (-0.02,-0.01)	<0.001	0 (-0.01,0)	0.081
Diabetes	0 (-0.01,0.01)	0.96	0 (-0.01,0)	0.16
Systolic blood pressure	-0.01 (-0.01,0)	0.0099	-0.01 (-0.01,0)	0.0087
Family history of MI	0 (-0.01,0)	0.53	0 (-0.01,0)	0.65
Dyslipidemia	0 (0,0.01)	0.25	0.01 (0,0.01)	0.0066
BMI	0.02 (0.01,0.03)	<0.001	0.02 (0.01,0.03)	<0.001
Socioeconomic status	0.01 (0,0.01)	0.028	0 (0,0.01)	0.12
**QTc Duration**				
Age*	5.26 (4.25,6.28)	<0.001	1.96 (0.93,2.99)	<0.001
Current smoking	-0.19 (-1.14,0.75)	0.69	-1.37 (-2.24,-0.49)	0.0023
Diabetes	1.32 (0.35,2.29)	0.0074	1.16 (0.28,2.05)	0.010
Systolic blood pressure*	4.9 (3.92,5.89)	<0.001	2.25 (1.27,3.23)	<0.001
Family history of MI	-0.78 (-1.7,0.15)	0.10	-0.73 (-1.58,0.13)	0.097
Dyslipidemia	0.61 (-0.33,1.54)	0.20	-0.35 (-1.23,0.54)	0.44
BMI*	4.41 (3.43,5.39)	<0.001	3.23 (2.31,4.15)	<0.001
Socioeconomic status	-0.05 (-1.01,0.91)	0.92	-0.84 (-1.76,0.08)	0.074

Regression coefficient per standard deviation (betas) and 95% confidence intervals [CI] are presented. Multivariable-adjustment comprised age, body mass index, systolic blood pressure, current smoking, Dyslipidemia, and a family history of MI. Intima-media thickness, left ventricular wall mass, PR interval, E/E’ and baseline pulse amplitude were log-transformed. FMD was squared-root transformed, presented model not adjusted for baseline diameter Heart failure definition comprised left ventricular ejection fraction. * Statistically significant sex interactions. MI stands for myocardial infarction, LEAD for lower extremity artery disease, PAT for peripipheral arterial tonometry.

Supplementary Table 3. Multivariable linear regressions for intermediate phenotypes adjusted for classical CVD risk factors in women younger than 51 years.

Variable	Women <51 years N=925	
	**Beta (95% CI)**	**P Value**
**Intima-media thickness**		
Age	0.04 (0.03,0.05)	<0.001
Current smoking	0.01 (0.01,0.02)	<0.001
Diabetes	0.01 (0,0.01)	0.075
Systolic blood pressure	0.02 (0.01,0.02)	<0.001
Family history of MI	0.01 (0,0.02)	0.0025
Dyslipidemia	0.01 (0,0.01)	0.058
BMI	0.01 (0,0.02)	0.0025
**Ankle-brachial index**		
Age	0 (-0.01,0.01)	0.59
Current smoking	-0.01 (-0.02,0)	0.022
Diabetes	-0.01 (-0.01,0)	0.097
Systolic blood pressure	0 (-0.01,0)	0.21
Family history of MI	0 (-0.01,0.01)	0.98
Dyslipidemia	0 (-0.01,0)	0.57
BMI	0.01 (0,0.01)	0.077
**Baseline brachial artery diameter**		
Age	0.06 (0.03,0.09)	<0.001
Current smoking	-0.01 (-0.04,0.02)	0.53
Diabetes	0 (-0.03,0.03)	0.92
Systolic blood pressure	0 (-0.03,0.04)	0.87
Family history of MI	-0.01 (-0.04,0.02)	0.46
Dyslipidemia	0.01 (-0.02,0.05)	0.36
BMI	0.15 (0.12,0.18)	<0.001
**Flow-mediated dilation**		
Age	-0.03 (-0.08,0.01)	0.12
Current smoking	0.01 (-0.03,0.06)	0.51
Diabetes	-0.02 (-0.06,0.02)	0.33
Systolic blood pressure	-0.03 (-0.08,0.01)	0.15
Family history of MI	-0.02 (-0.06,0.02)	0.34
Dyslipidemia	-0.03 (-0.07,0.02)	0.21
BMI	-0.07 (-0.11,-0.03)	0.0016
**Baseline pulse amplitude**		

Variable	Women <51 years N=925	
	**Beta (95% CI)**	**P Value**
Age	0.1 (0.04,0.15)	<0.001
Current smoking	0.09 (0.04,0.14)	0.0013
Diabetes	-0.01 (-0.07,0.04)	0.63
Systolic blood pressure	-0.1 (-0.16,-0.04)	<0.001
Family history of MI	-0.03 (-0.09,0.02)	0.21
Dyslipidemia	0.08 (0.02,0.13)	0.0062
BMI	0.24 (0.19,0.3)	<0.001
**Peripheral arterial tonometry ratio**		
Age	0 (-0.02,0.03)	0.90
Current smoking	-0.02 (-0.05,0)	0.047
Diabetes	0.01 (-0.02,0.03)	0.66
Systolic blood pressure	0.07 (0.05,0.1)	<0.001
Family history of MI	0.02 (-0.01,0.04)	0.15
Dyslipidemia	-0.04 (-0.06,-0.01)	0.0021
BMI	-0.08 (-0.1,-0.05)	<0.001
**Left ventricular ejection fraction**		
Age	0.6 (0.23,0.96)	0.0012
Current smoking	-0.06 (-0.41,0.29)	0.75
Diabetes	0.23 (-0.12,0.58)	0.20
Systolic blood pressure	-0.07 (-0.44,0.3)	0.70
Family history of MI	-0.25 (-0.6,0.1)	0.16
Dyslipidemia	0.12 (-0.23,0.47)	0.51
BMI	-0.27 (-0.63,0.09)	0.15
**Left ventricular wall mass**		
Age	0 (-0.01,0.02)	0.49
Current smoking	0.01 (0,0.03)	0.032
Diabetes	0 (-0.01,0.01)	0.94
Systolic blood pressure	0.04 (0.02,0.05)	<0.001
Family history of MI	0.01 (-0.01,0.02)	0.31
Dyslipidemia	-0.01 (-0.02,0.01)	0.37
BMI	0.11 (0.1,0.12)	<0.001
**E/E’**		
Age	0.04 (0.02,0.05)	<0.001
Current smoking	0.02 (0.01,0.04)	0.0044
Diabetes	0.02 (0,0.03)	0.025
Systolic blood pressure	0.03 (0.02,0.05)	<0.001

Variable	Women <51 years N=925	
	**Beta (95% CI)**	**P Value**
Family history of MI	0.01 (0,0.03)	0.068
Dyslipidemia	0.01 (0,0.02)	0.18
BMI	0.03 (0.02,0.05)	<0.001
**PR interval**		
Age	0.01 (0.01,0.02)	0.0013
Current smoking	-0.01 (-0.01,0)	0.21
Diabetes	-0.01 (-0.02,0)	0.16
Systolic blood pressure	-0.01 (-0.02,0)	0.17
Family history of MI	0 (-0.01,0.01)	0.94
Dyslipidemia	0.01 (0,0.02)	0.11
BMI	0.01 (0,0.02)	0.0025
**QTc Duration**		
Age	-0.66 (-2,0.68)	0.33
Current smoking	-2.2 (-3.51,-0.9)	<0.001
Diabetes	1.12 (-0.18,2.42)	0.091
Systolic blood pressure	2.43 (1.06,3.79)	<0.001
Family history of MI	-0.98 (-2.28,0.32)	0.14
Dyslipidemia	0.55 (-0.76,1.86)	0.41
BMI	2.07 (0.74,3.41)	0.0023

Regression coefficients per standard deviation increase (betas) and 95%-confidence intervals (95%-CI) are presented. Multivariable-adjustment comprised age, body mass index, systolic blood pressure, current smoking, Dyslipidemia, and a family history of MI. Intima-media thickness, left ventricular wall mass, PR interval, and baseline pulse amplitude were log-transformed. Flow-mediated dilation was squared-root transformed, presented model not adjusted for baseline diameter

BMI stands for body mass index, MI for myocardial infarction

Reference List

1. Breiman L. Random Forests. Machine Learning (2001) 45:5-32.
