# Supplementary figures and images for: Sex Differences in Correlates of Intermediate Phenotypes and Prevalent Cardiovascular Disease in the General Population
Source: Front Cardiovasc Med. 2015 Apr 15;2:15. doi: 10.3389/fcvm.2015.00015 (PMC4671364; doi:10.3389/fcvm.2015.00015)

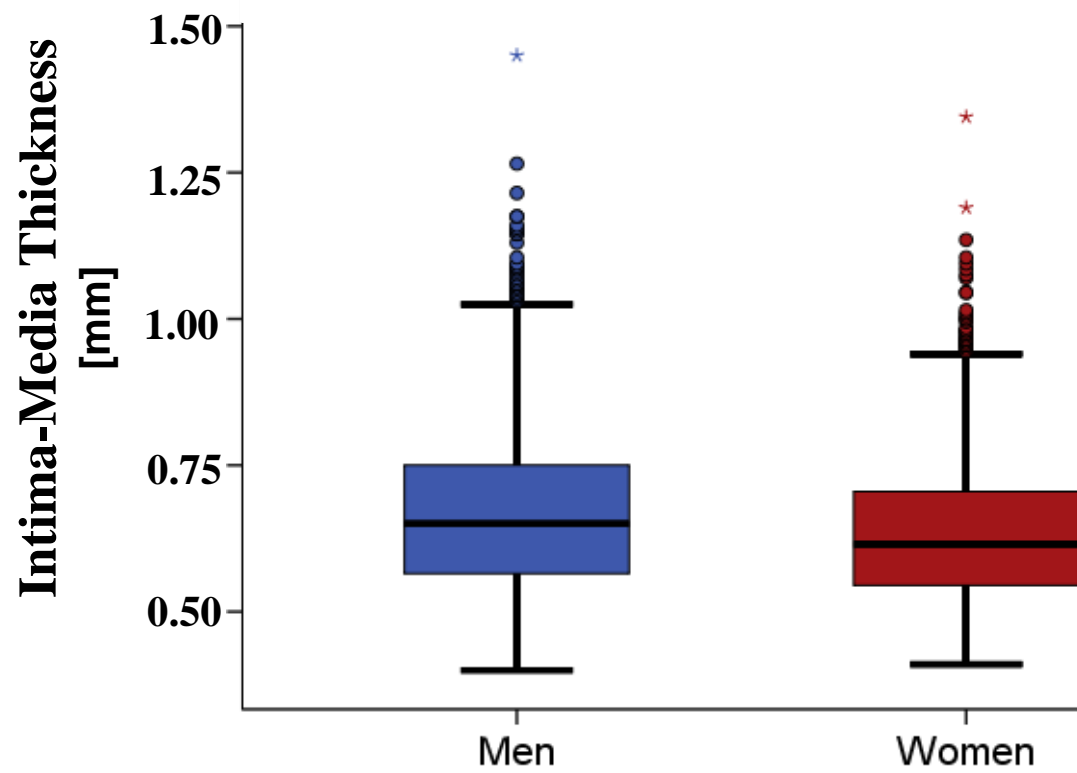

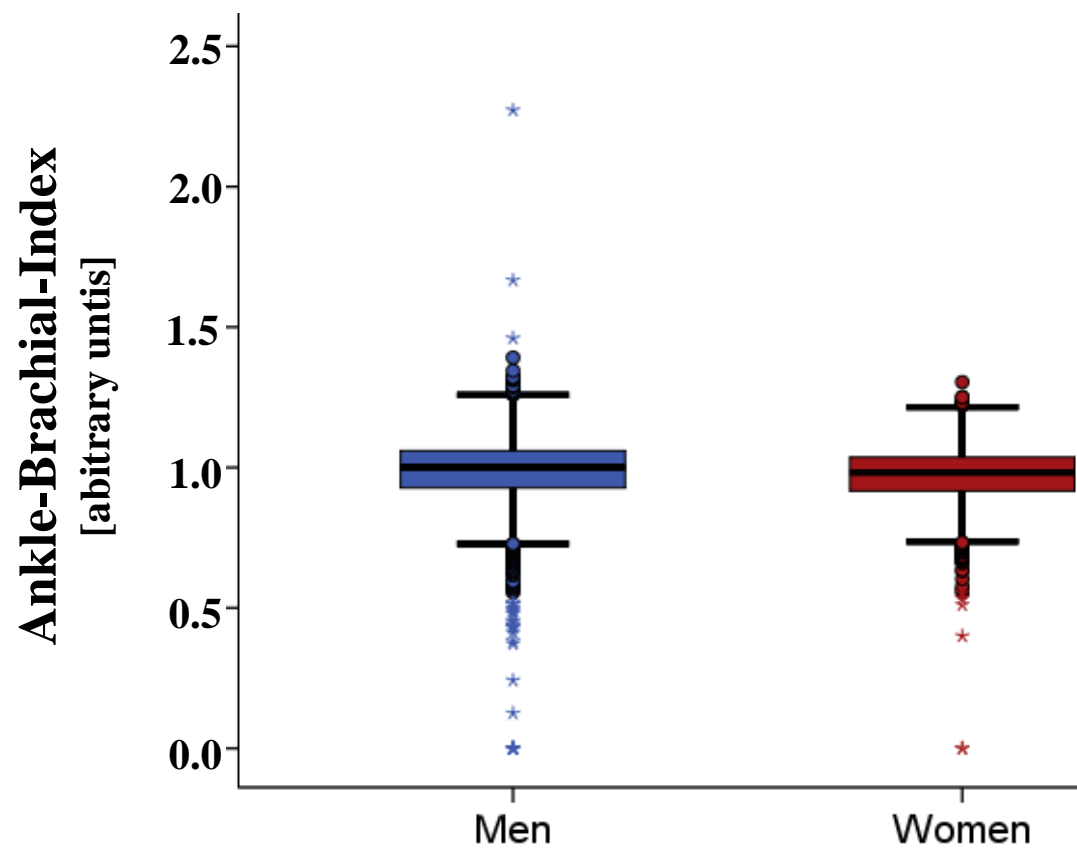

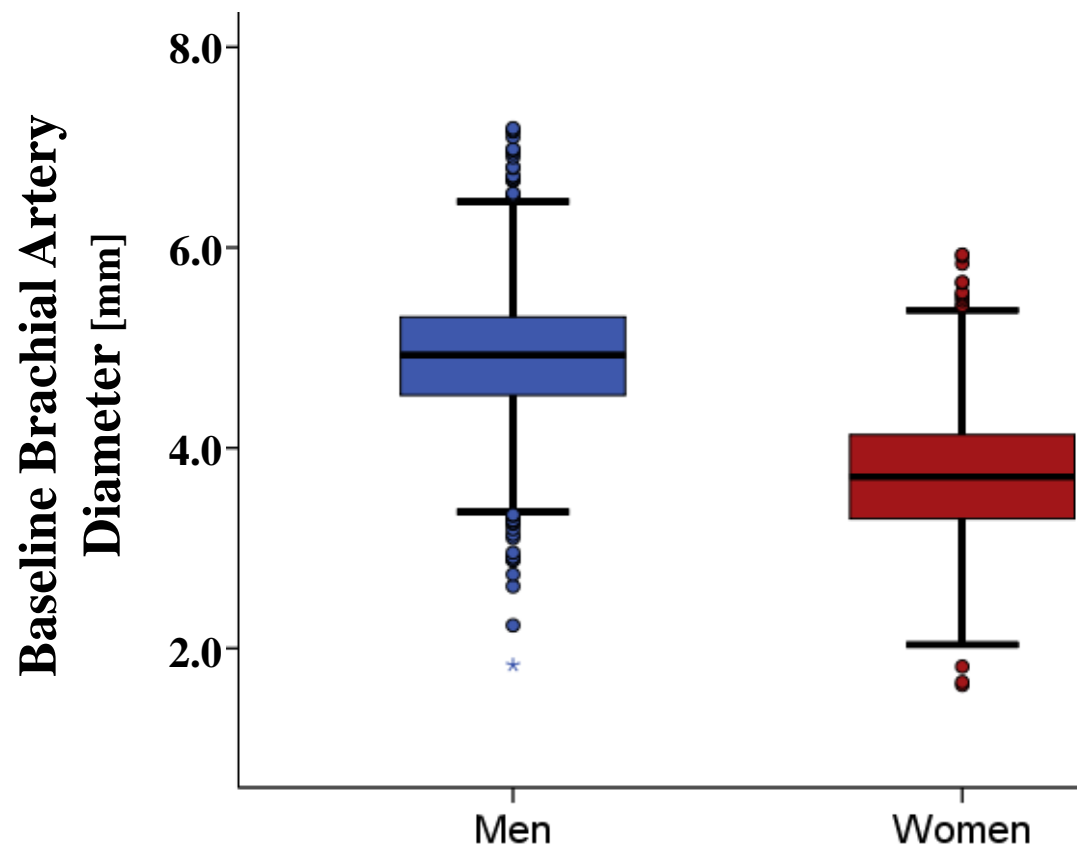

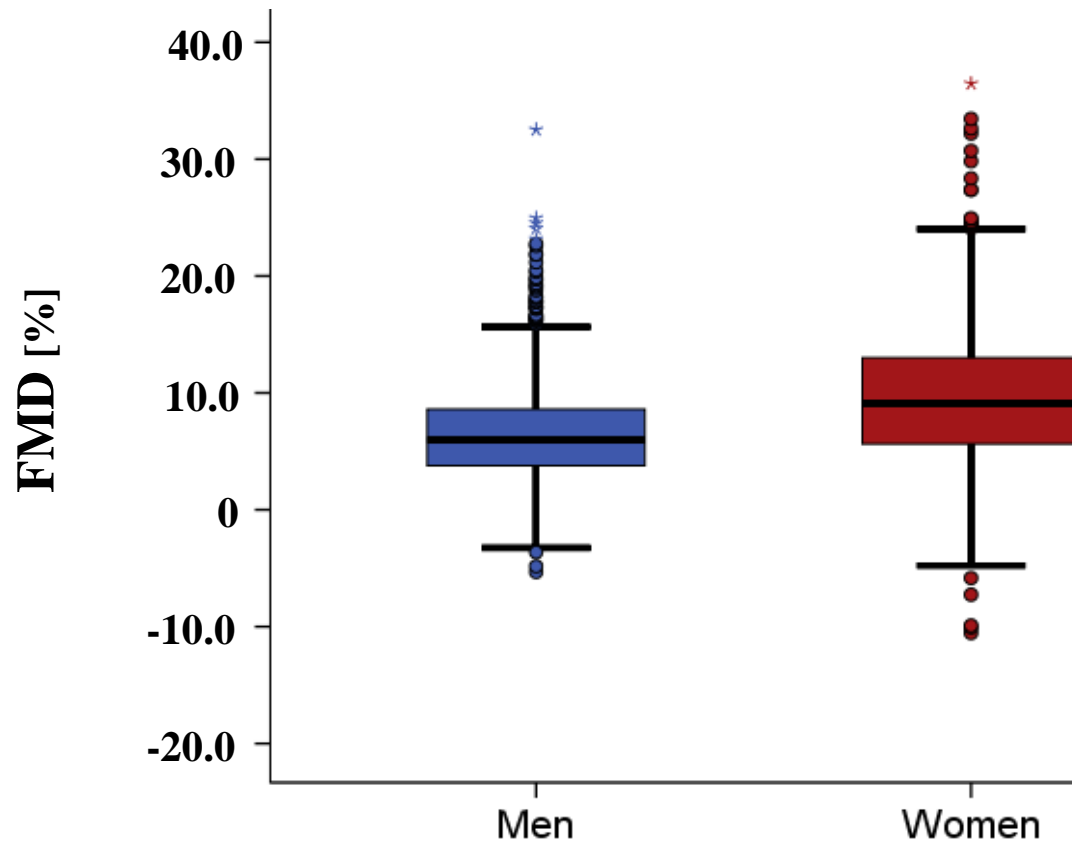

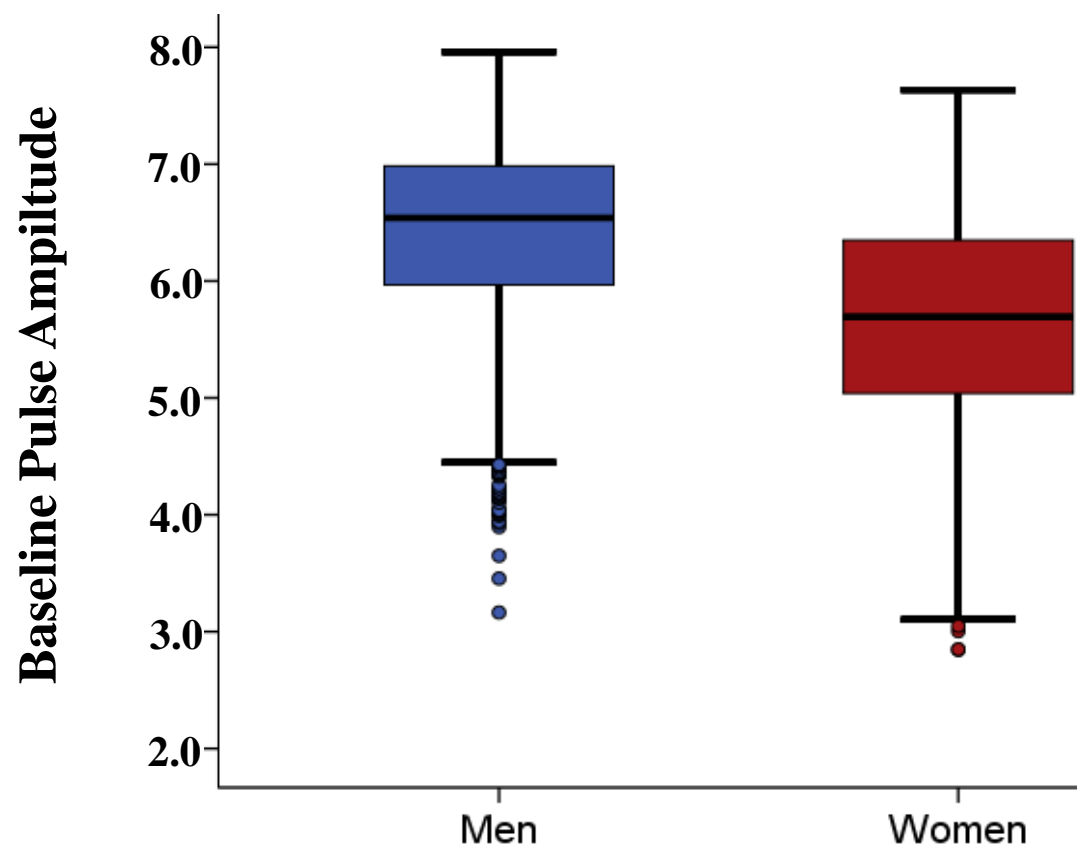

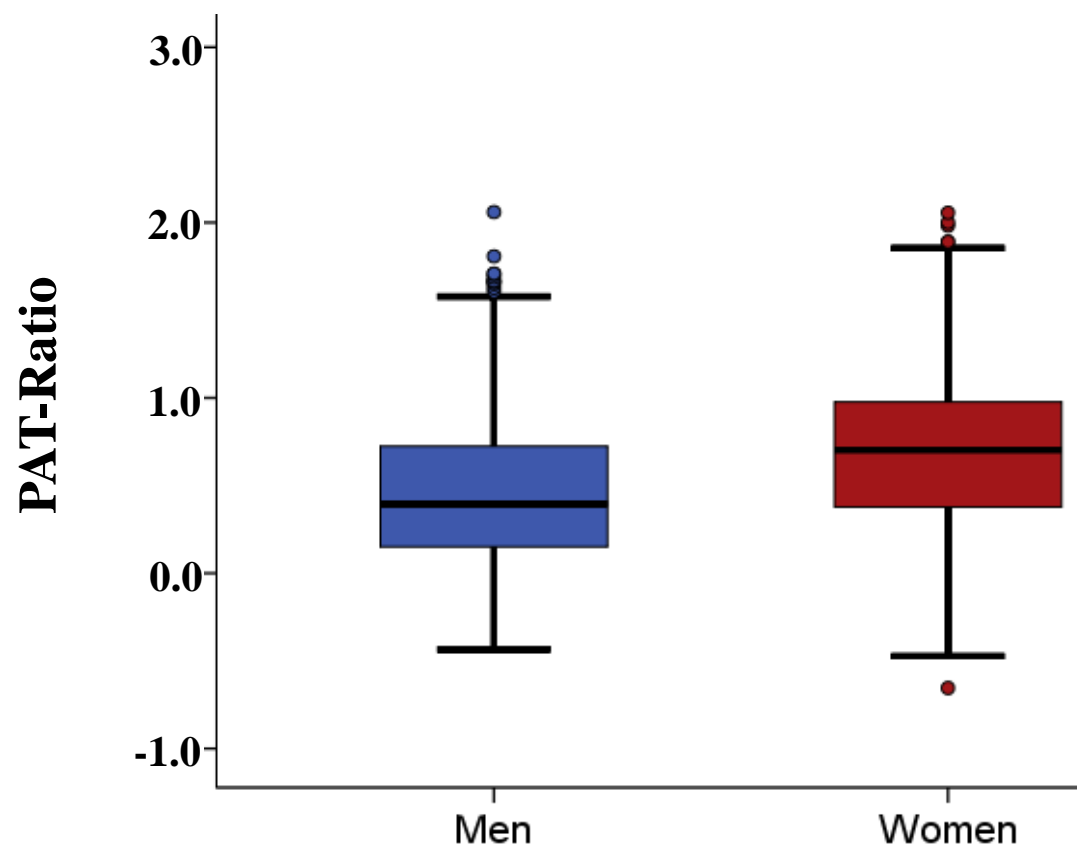

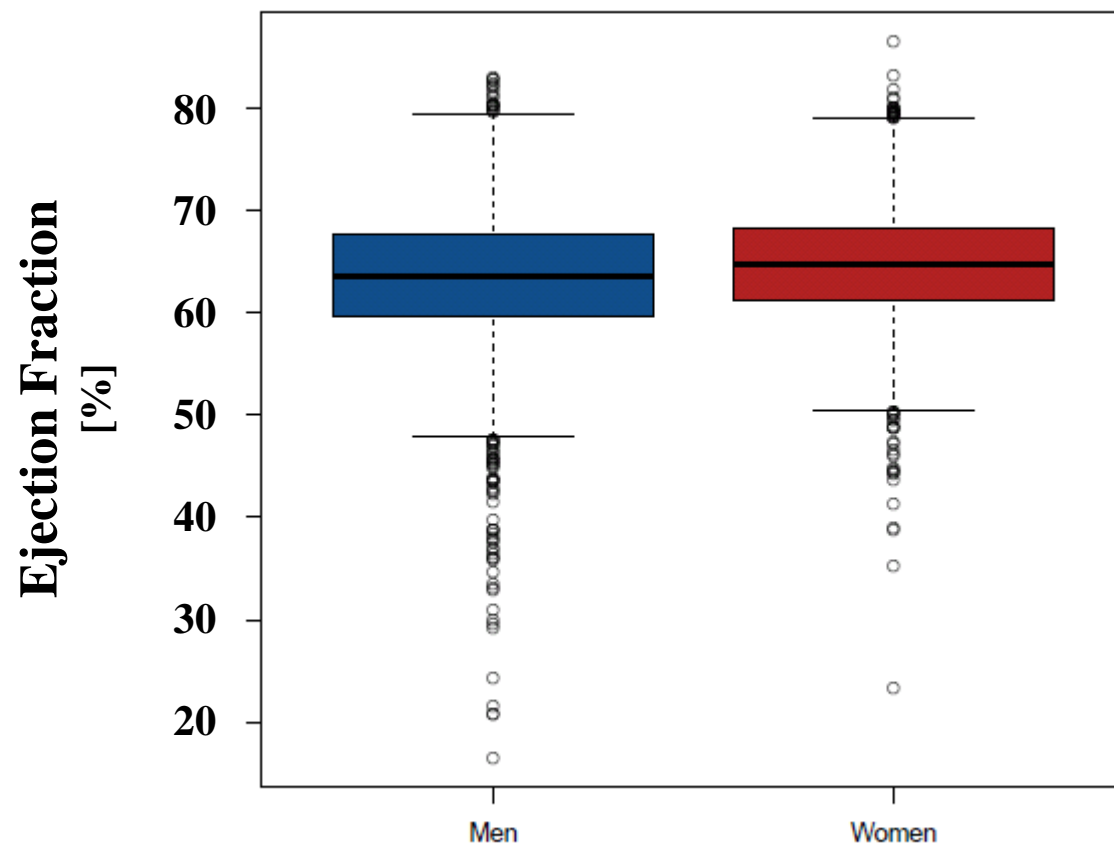

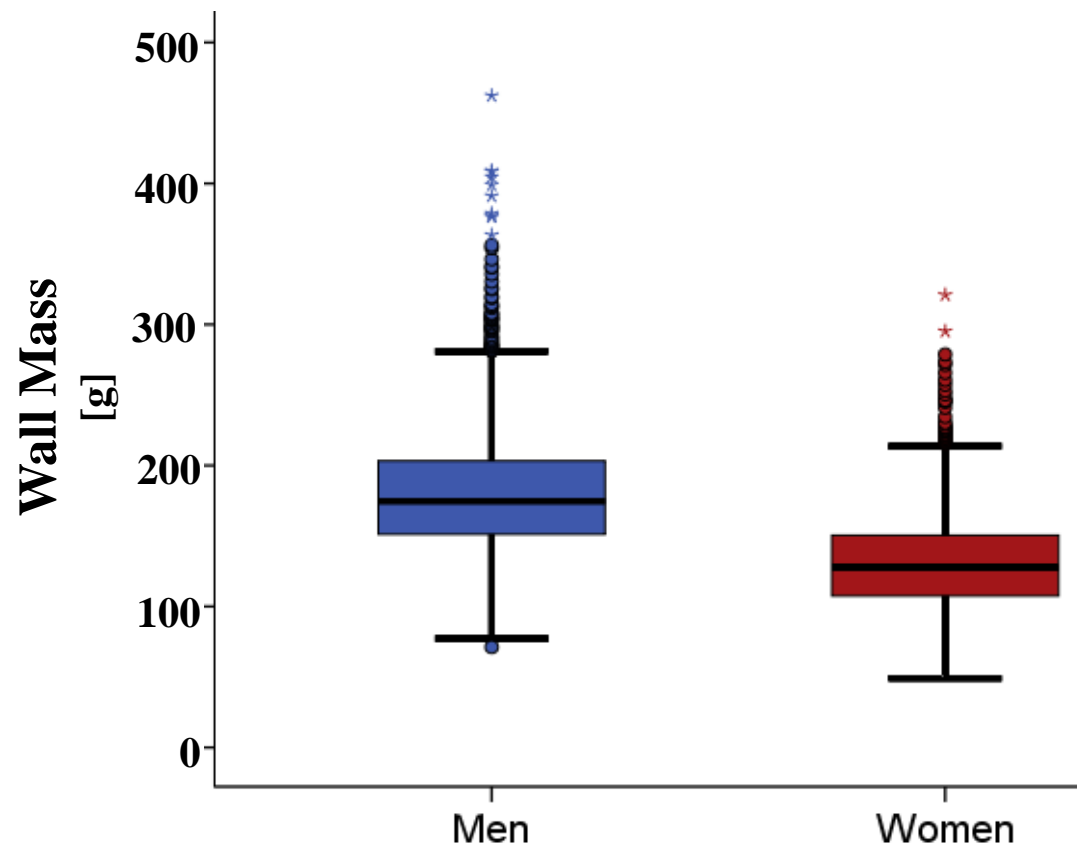

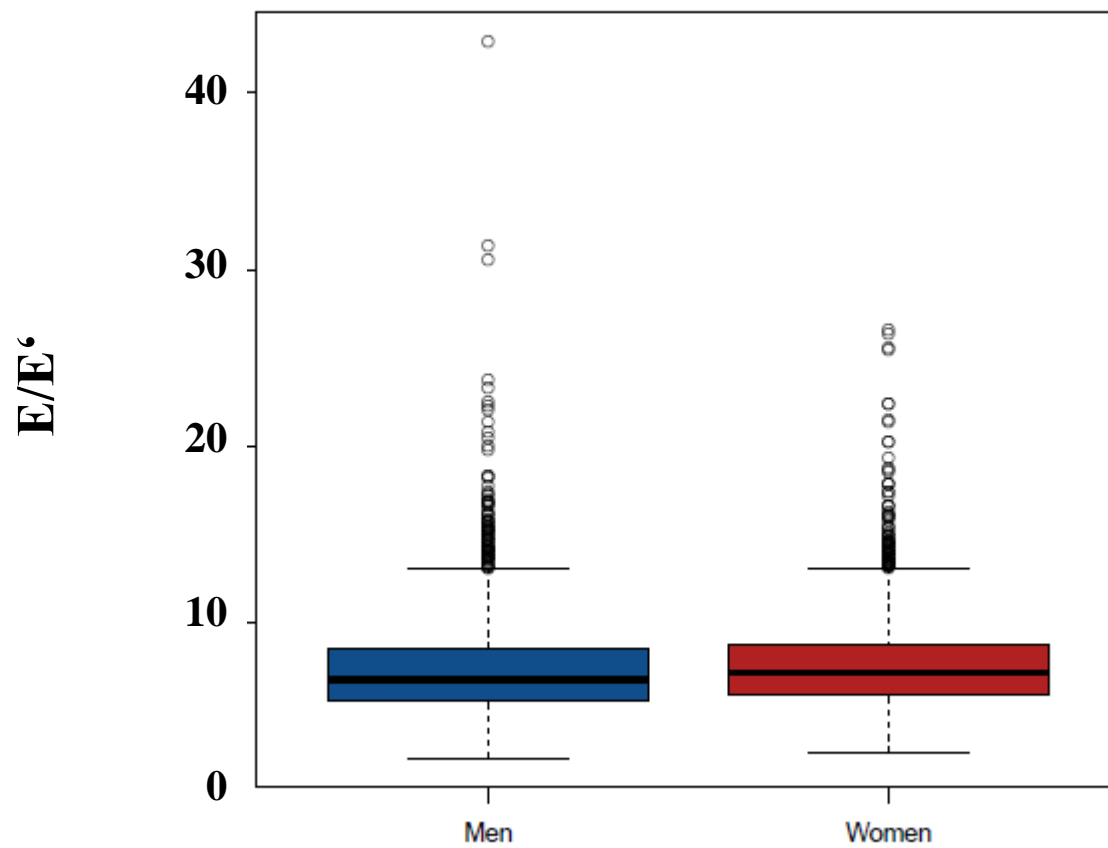

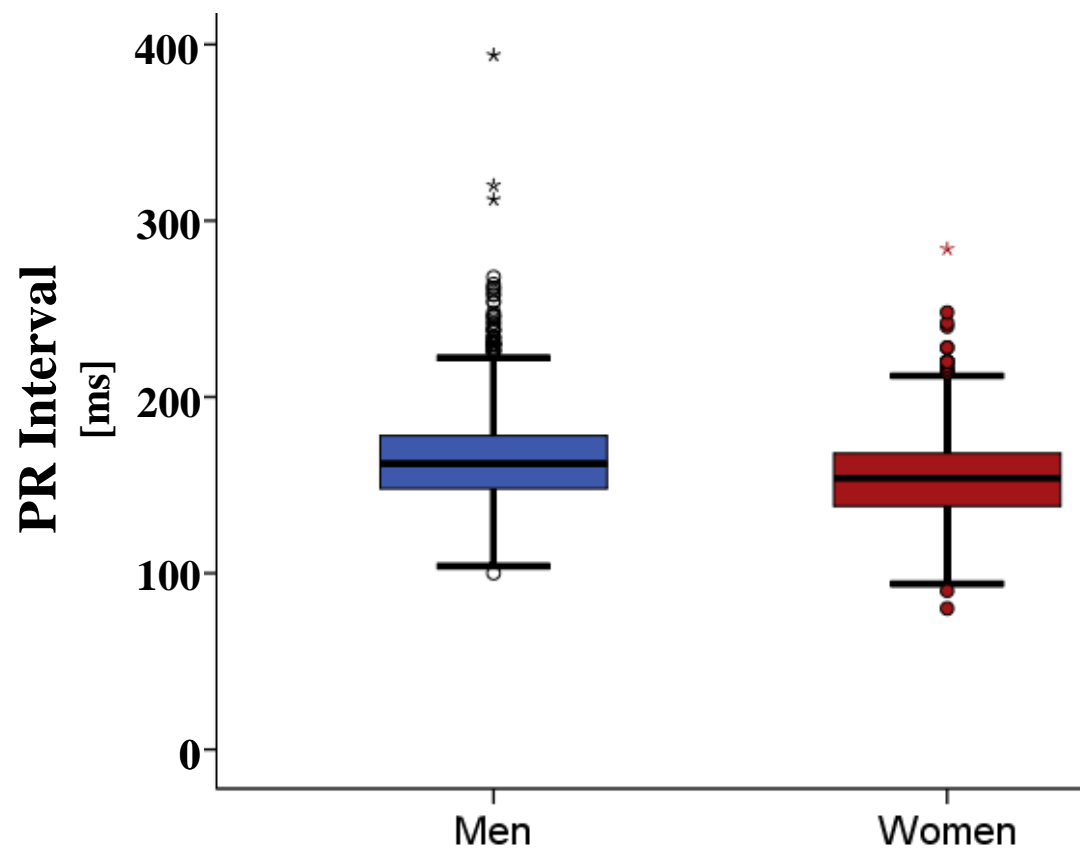

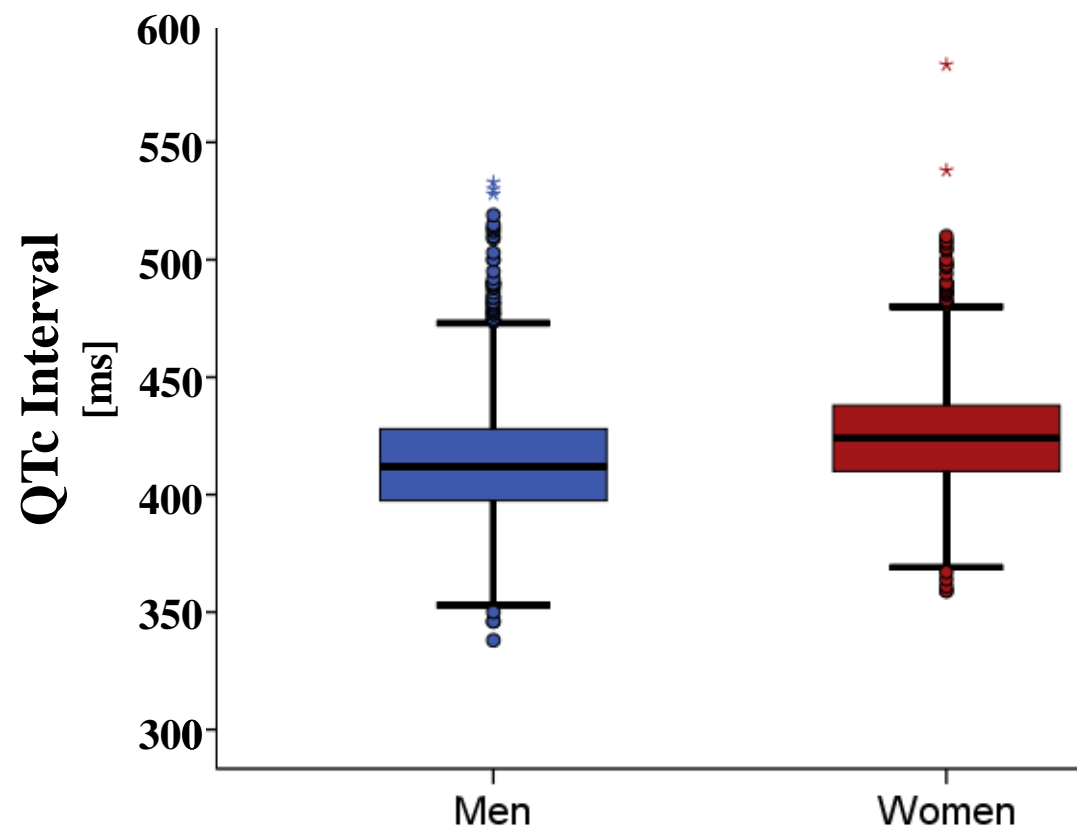

Supplement: Supplementary file 2 [file Image_1.PDF]
