# Supplementary material for: Sex Differences in Correlates of Intermediate Phenotypes and Prevalent Cardiovascular Disease in the General Population
Source: Front Cardiovasc Med. 2015 Apr 15;2:15. doi: 10.3389/fcvm.2015.00015 (PMC4671364; doi:10.3389/fcvm.2015.00015)

Intima-media thickness

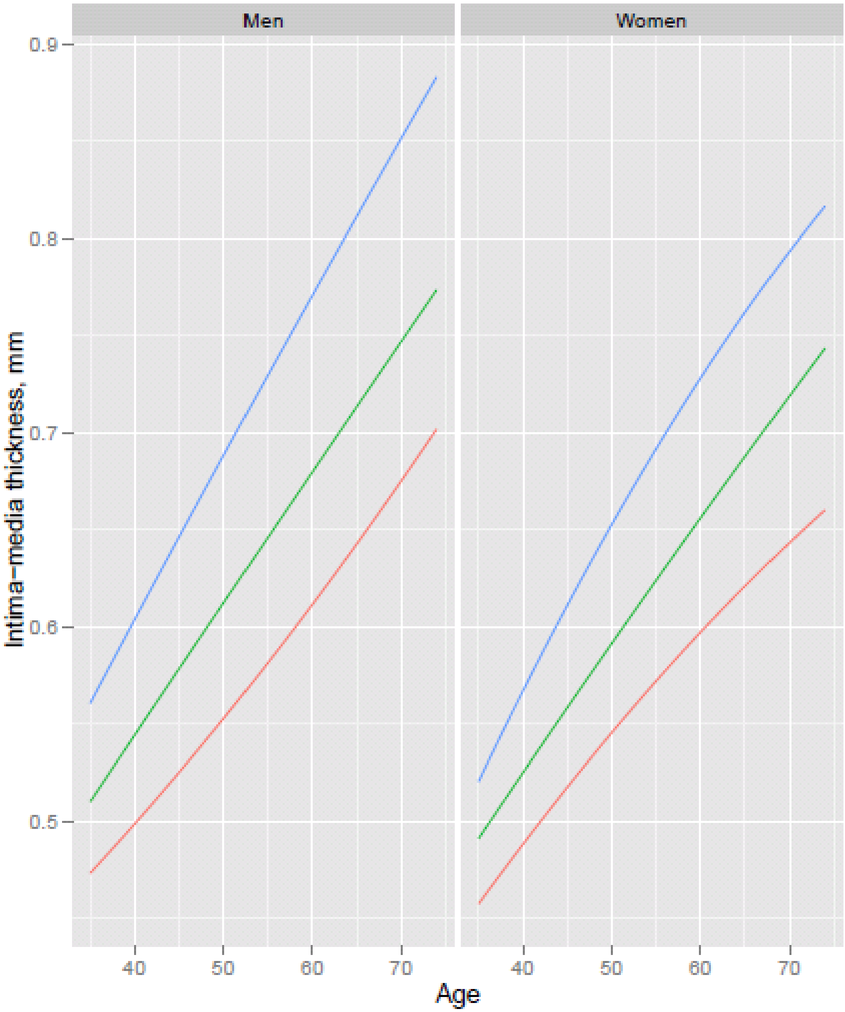

Ankle-brachial index

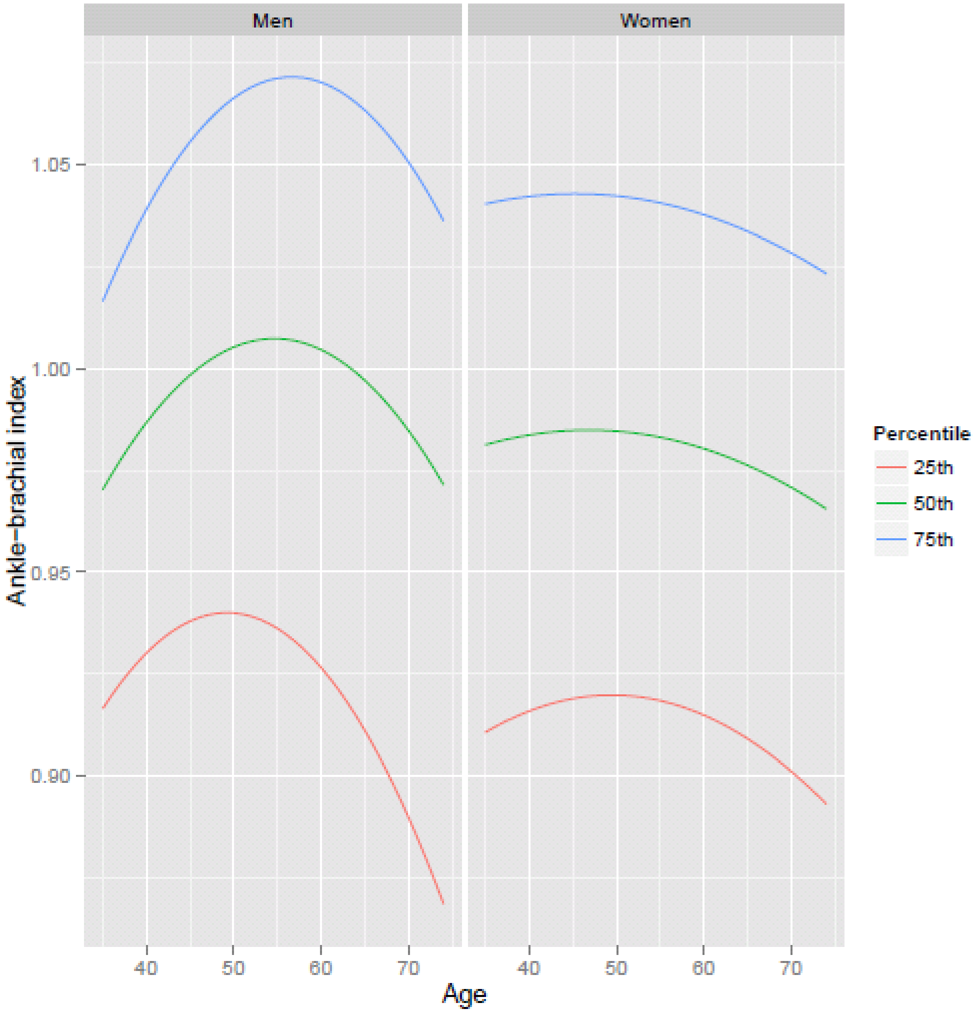

Baseline brachial artery diameter

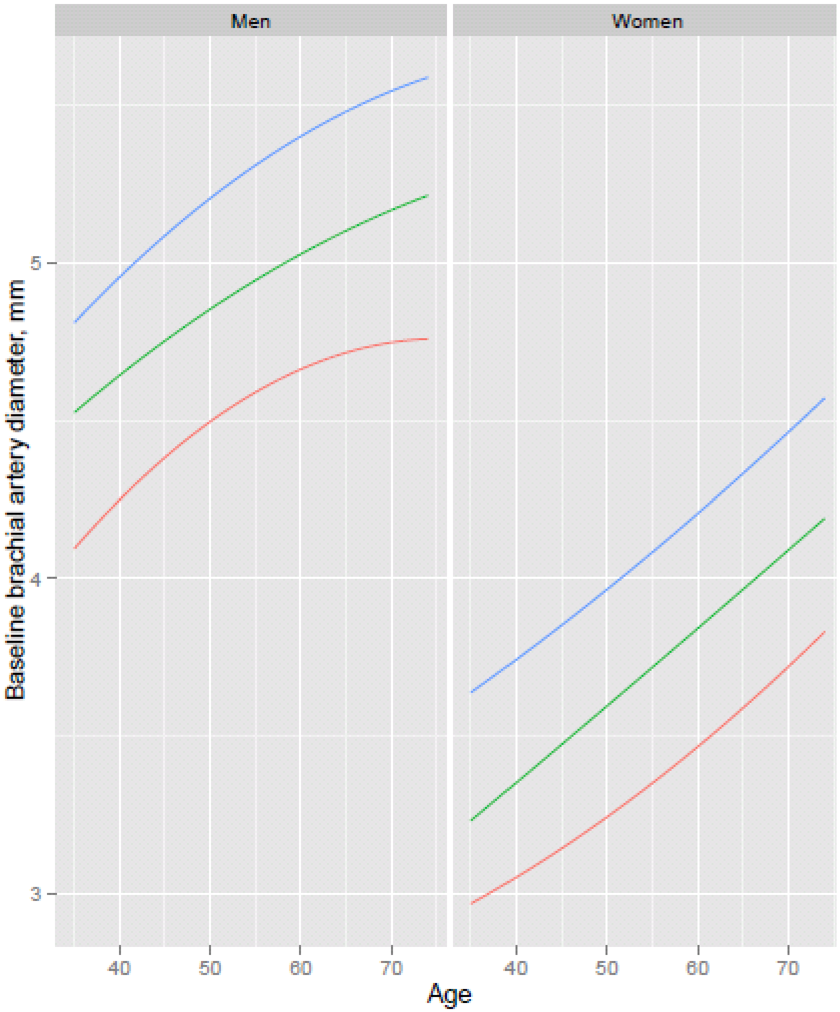

FMD

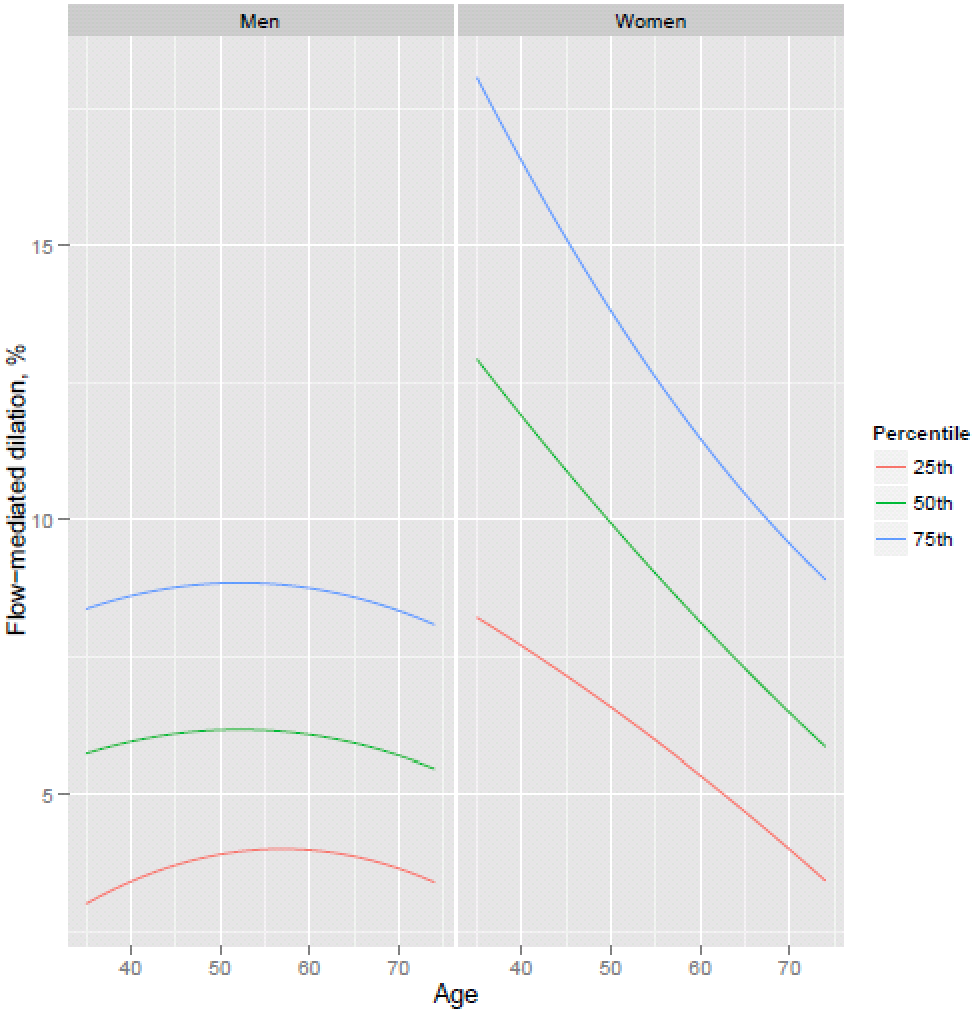

Baseline pulse amplitude

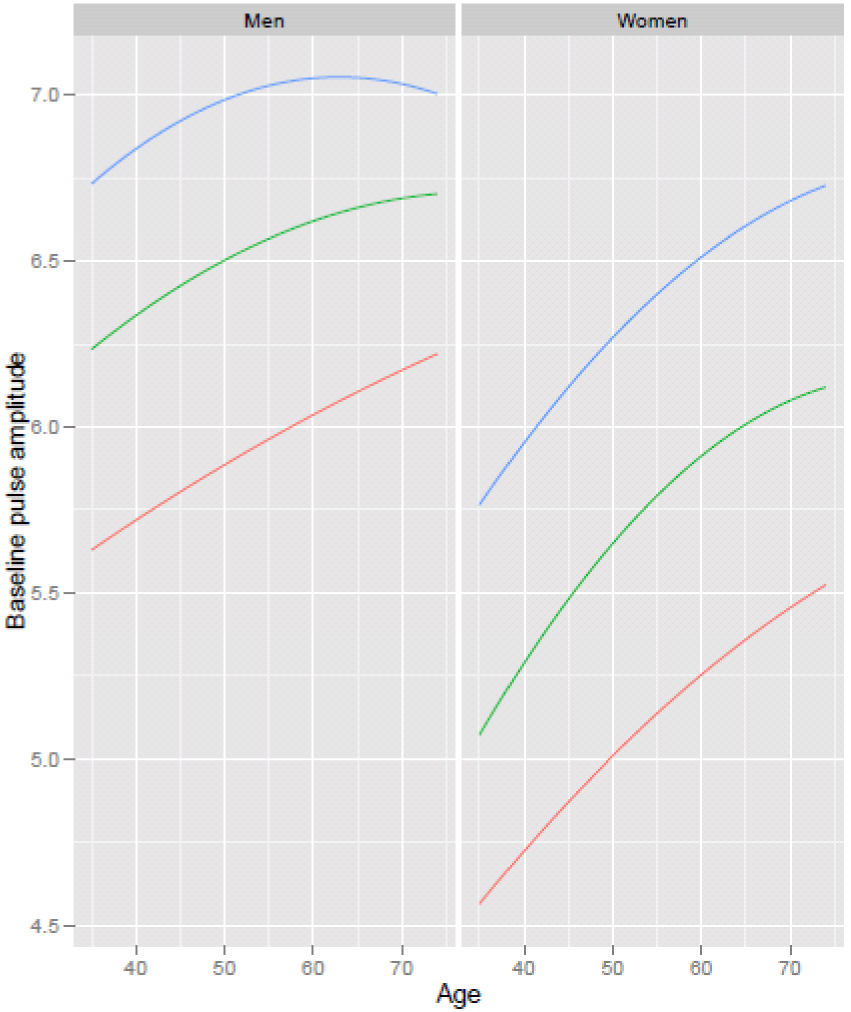

PAT-ratio

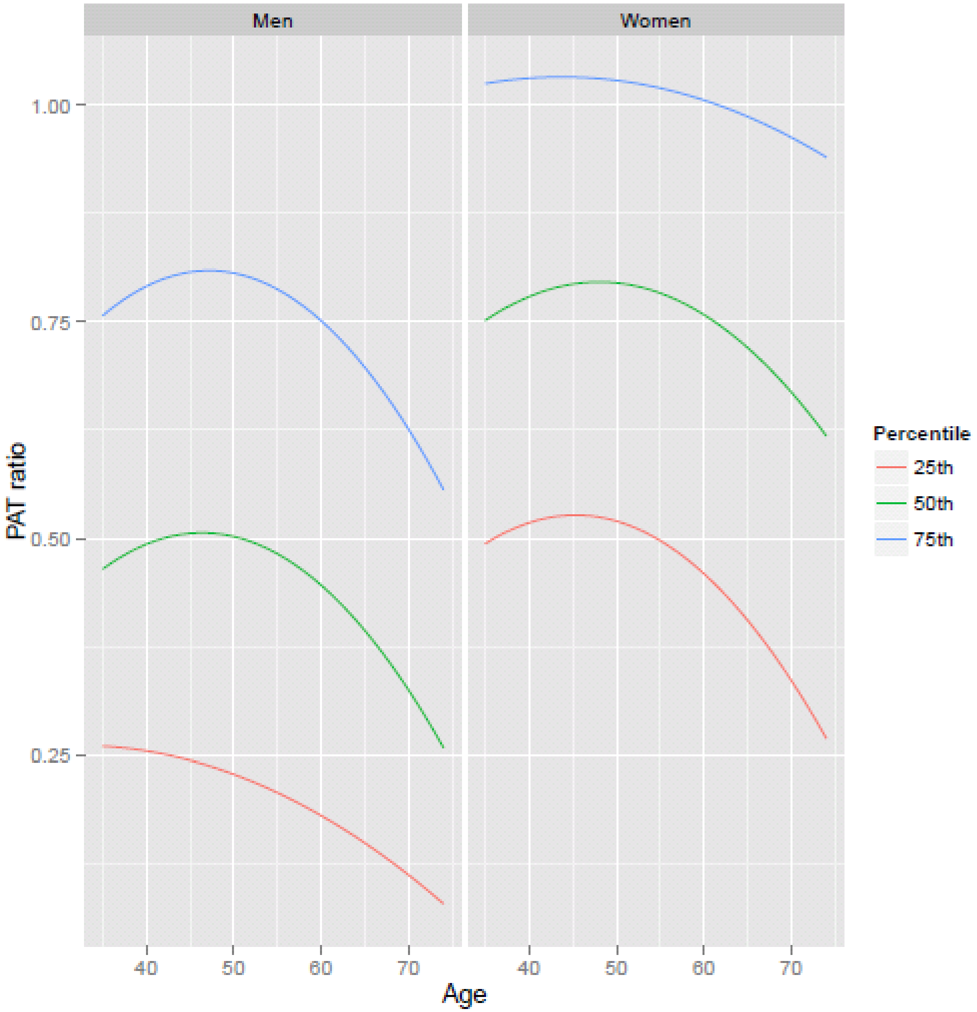

Left ventricular ejection fraction

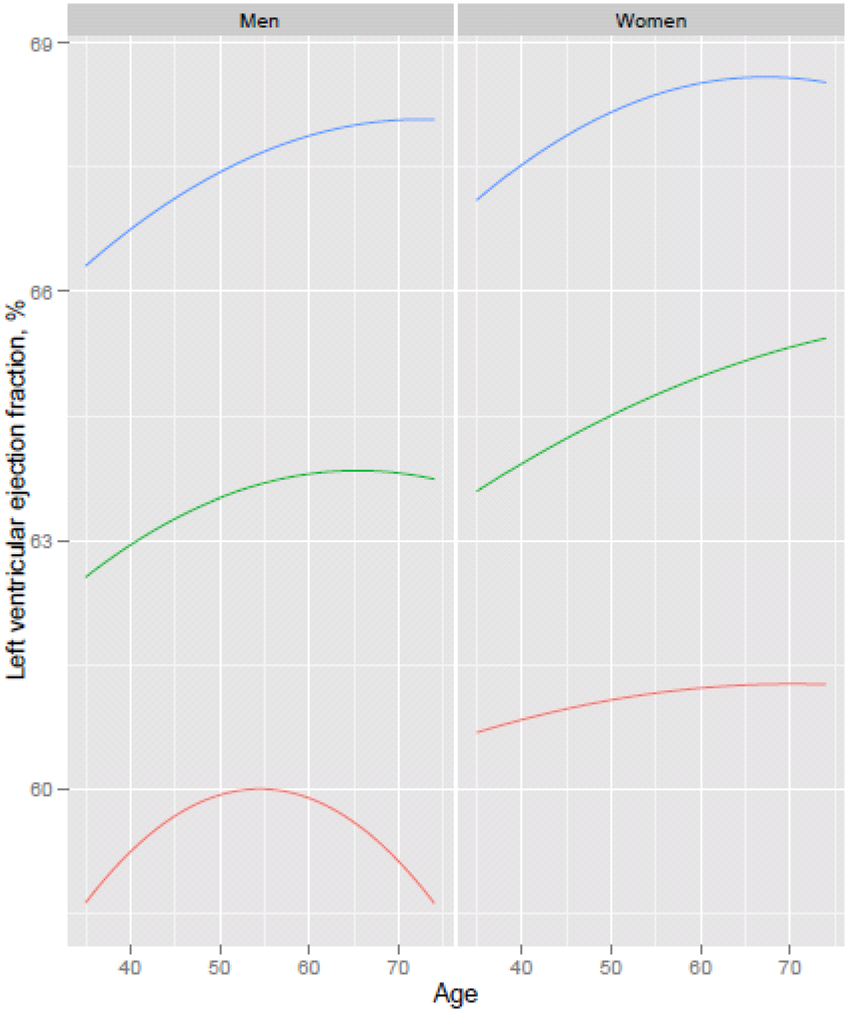

Left ventricular wall mass

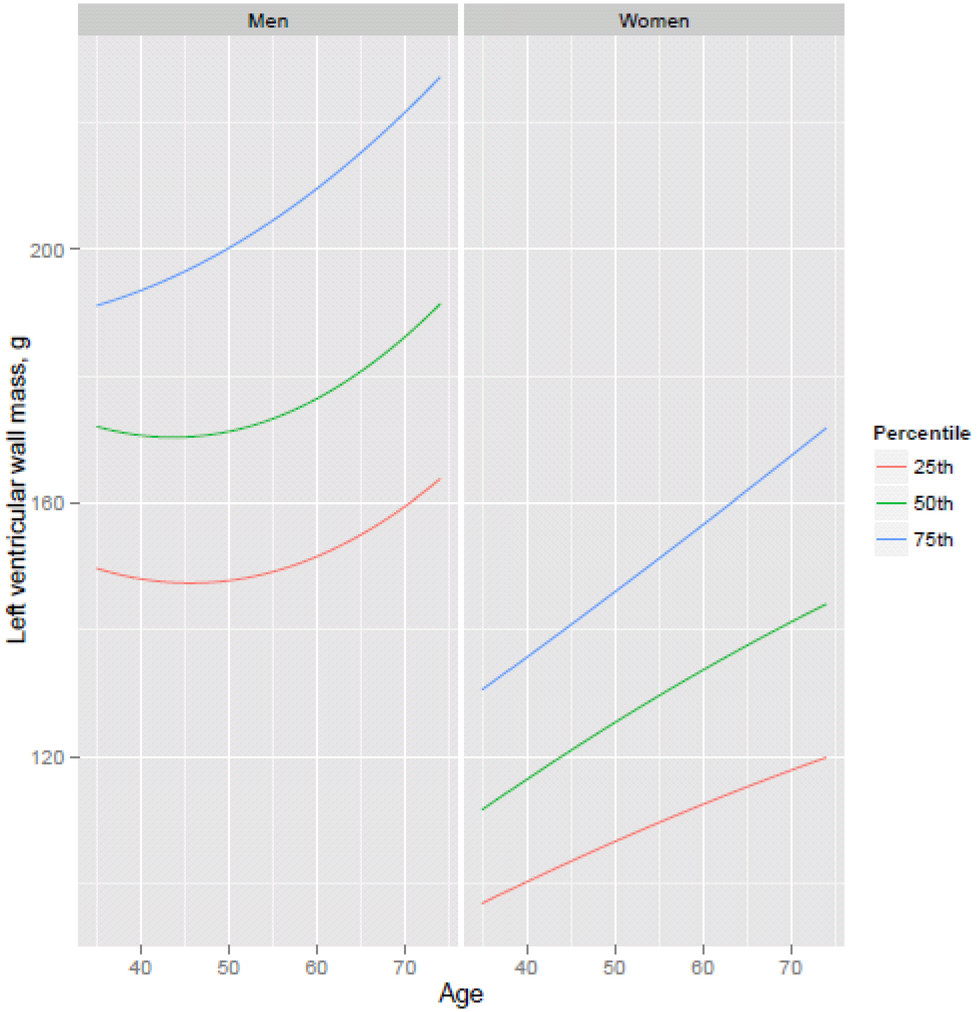

E/E‘

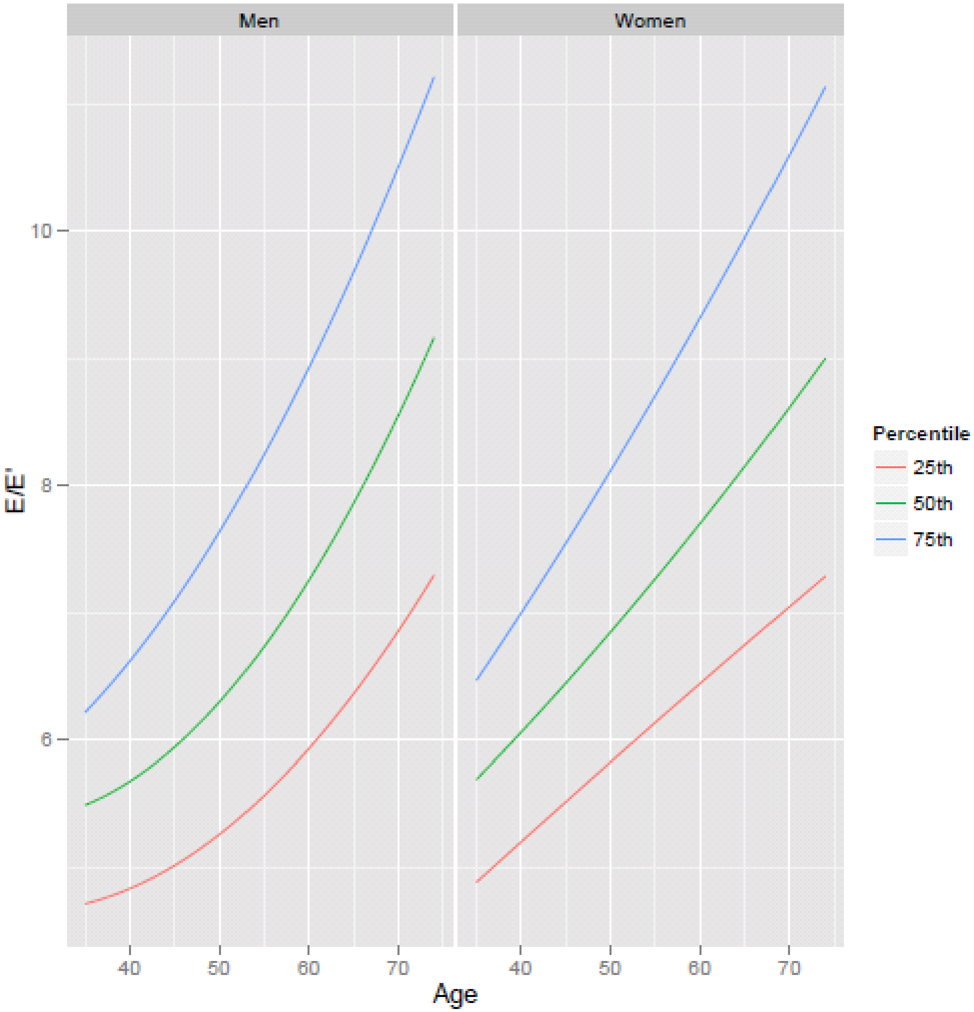

Men
Women
10
8
6
E/E'
40
50
60
70
40
50
60
70
Age
Percentile
25th
50th
75th

PR interval

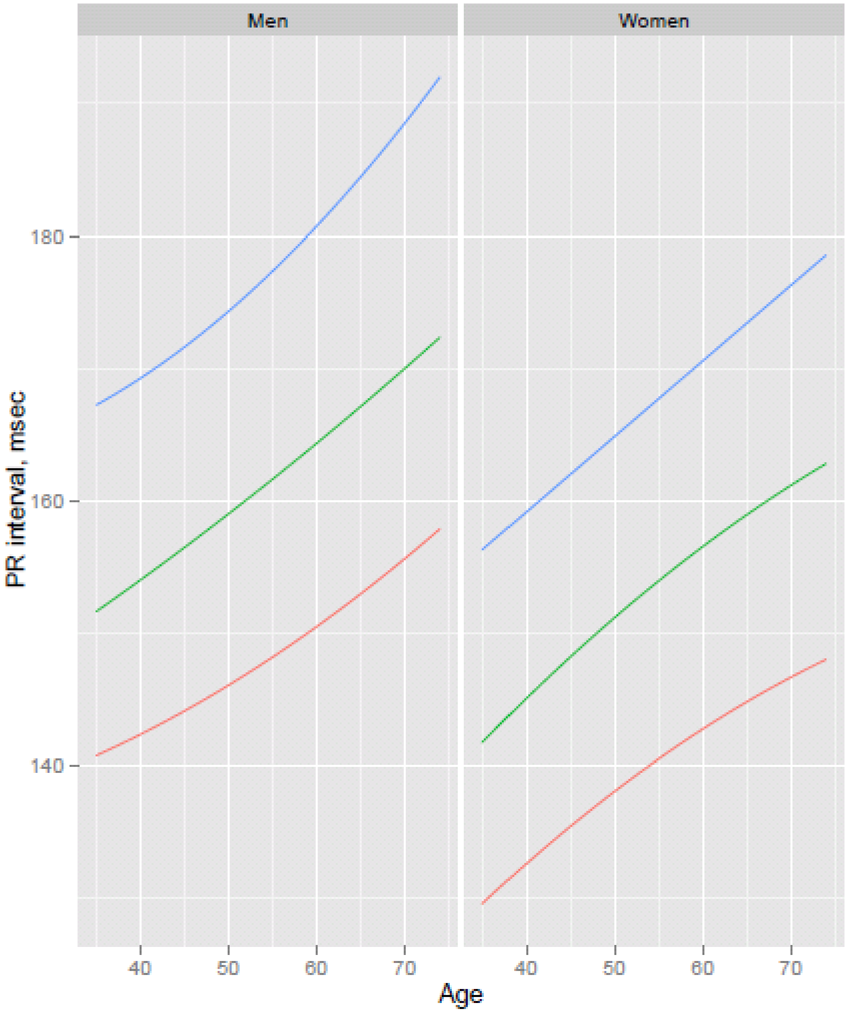

QTc duration

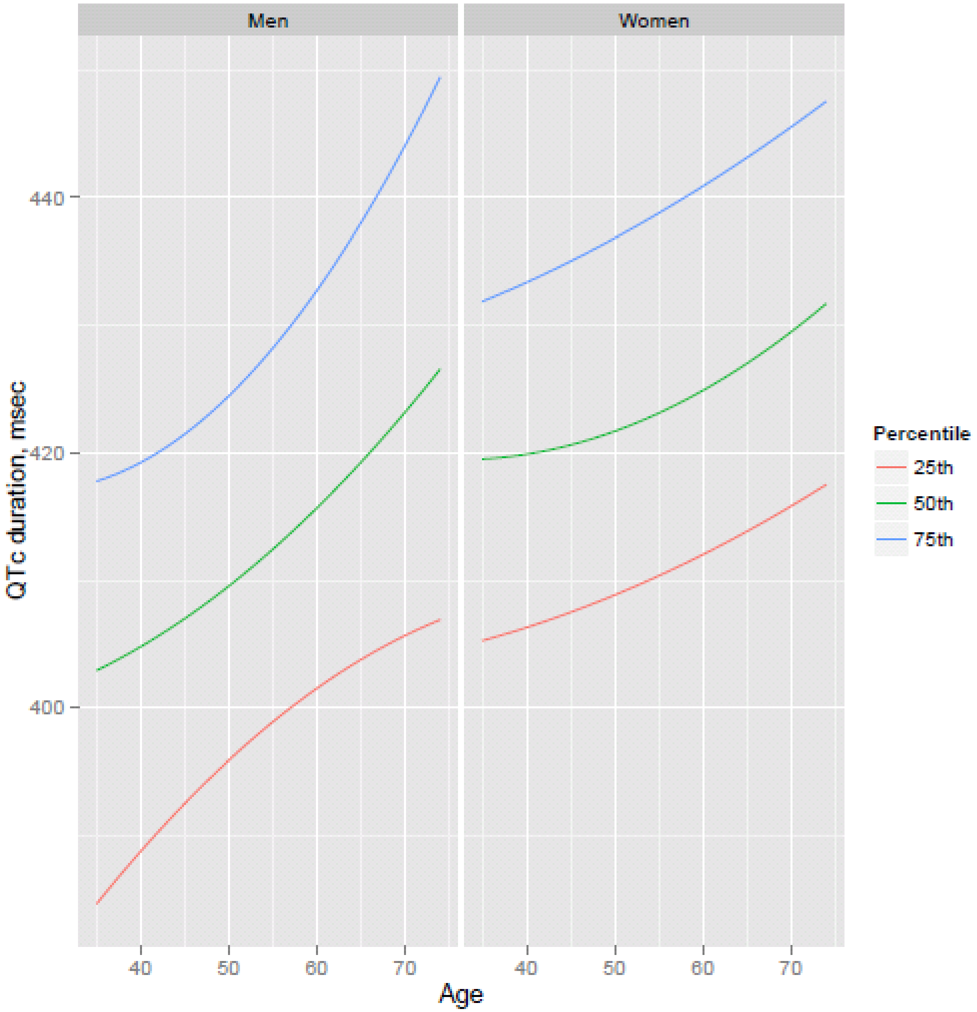

Supplement: Supplementary file 3 [file Image_2.PDF]
